# Multiple haplotype-resolved genomes reveal population patterns of gene and protein diplotypes

**DOI:** 10.1038/ncomms6569

**Published:** 2014-11-26

**Authors:** Margret R. Hoehe, George M. Church, Hans Lehrach, Thomas Kroslak, Stefanie Palczewski, Katja Nowick, Sabrina Schulz, Eun-Kyung Suk, Thomas Huebsch

**Affiliations:** 1 Department of Vertebrate Genomics, Max Planck Institute for Molecular Genetics, Berlin D-14195, Germany; 2 Department of Genetics, Harvard Medical School, Boston, Massachusetts 02115, USA; 3 Department of Computer Science, University of Leipzig, Leipzig D-04107, Germany; 4 These authors contributed equally to this work

## Abstract

To fully understand human biology and link genotype to phenotype, the phase of DNA variants must be known. Here we present a comprehensive analysis of haplotype-resolved genomes to assess the nature and variation of haplotypes and their pairs, diplotypes, in European population samples. We use a set of 14 haplotype-resolved genomes generated by fosmid clone-based sequencing, complemented and expanded by up to 372 statistically resolved genomes from the 1000 Genomes Project. We find immense diversity of both haploid and diploid gene forms, up to 4.1 and 3.9 million corresponding to 249 and 235 per gene on average. Less than 15% of autosomal genes have a predominant form. We describe a ‘common diplotypic proteome’, a set of 4,269 genes encoding two different proteins in over 30% of genomes. We show moreover an abundance of *cis* configurations of mutations in the 386 genomes with an average *cis*/*trans* ratio of 60:40, and distinguishable classes of *cis*- versus *trans*-abundant genes. This work identifies key features characterizing the diplotypic nature of human genomes and provides a conceptual and analytical framework, rich resources and novel hypotheses on the functional importance of diploidy.

## Abstract (web summary)

Knowing which genetic variants exist on either parental chromosome requires diploid human genomes to be phased. Here the authors generate haplotype-resolved genomes and identify a large diversity of haploid and diploid gene forms, a common diplotypic proteome, and an abundance of *cis* configurations of mutations, highlighting the functional importance of diploidy.

## Graphical abstract

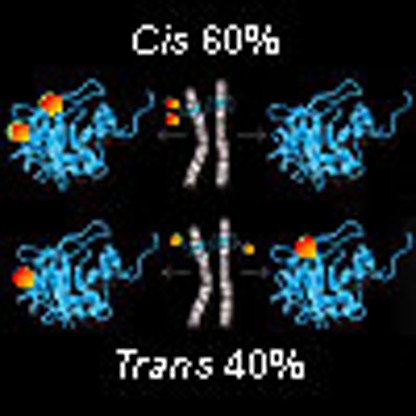

Human genomes are diploid by nature. In an ideal world without technical limitations, we would approach genome analysis by reading both the maternal and paternal sequences independently. We would determine which homologous genes, proteins and regulatory sequences were the same or different^1^, and distinguish the latter by their unique combinations of variants. Thus, beyond variants, we would catalogue and characterize their haplotypes and pairs thereof, diplotypes, representing functional units. To link genetic variation to gene function and phenotype, we would distinguish diplotypes further by their specific configurations of mutations. *Cis* configurations with two or more mutations on the same chromosome, for example, leave one form of the gene unperturbed, while *trans* configurations with mutations on both chromosomes may affect both forms of the gene^2^. Transcriptomes would be defined by expression of either one or both parental homologues in spatiotemporal and environmental context. The functionally active genome would be viewed as the result of the specific haploid or diploid protein forms interacting in genome-wide networks.

While tens of thousands of human genomes have been read out as ‘mixed diploid’ sequences to date, just over a dozen have been molecularly haplotype resolved^3^,^4^,^5^,^6^,^7^,^8^ and reported mostly with a technical focus. The diplotypic nature of the human genome and its potential functional implications have, however, barely been addressed. With our previous work, we have generated a virtually completely haplotype-resolved genome, ‘Max Planck One’ (MP1)^4^ and performed dissection of an individual’s ‘diplotype’^9^: determined the molecular diplotypes encoding 17,861 autosomal genes at the sequence and protein level; assessed the *cis* versus *trans* configurations of perturbing mutations; annotated *cis* and *trans* in relation to gene function and disease and examined the occurrence of protein diplotypes in pathways and ‘haploid landscapes’^10^.

Here we present a first systematic analysis of diplotype architecture at the population level. As a starting point, we describe a new set of 12 molecularly haplotype-resolved European genomes. With MP1 and NA12878 resolved by us previously^4^,^5^, an unprecedented set of 14 molecularly phased genomes laid the foundation for our analyses, complemented and expanded by up to 372 statistically resolved genomes of European descent from the 1000 Genomes Project (1000G)^11^. With the analysis of multiple haplotype-resolved genomes we aimed to get a clearer picture of the ‘true’ molecular toolbox underlying cellular and organismal processes and their variation in a population. Moreover, we aimed to extract common features and principles characterizing diploid gene and genome function. We addressed the following specific objectives: (i) to determine the entirety of different gene and protein haplotypes and diplotypes in the European population, and evaluate their frequencies of occurrence (FoO); (ii) to examine whether certain classes of genes preferentially encode two different forms of the protein to gain insight into the potential functional importance of diploidy and (iii) to evaluate the distribution of *cis* versus *trans* configurations of mutations at the gene and whole-genome level to uncover common patterns of phase.

In summary, our analysis of multiple haplotype-resolved genomes reveals a large diversity of haploid and diploid gene forms, in the range of several millions in 386 genomes, with the vast majority of genes lacking a predominant form. This diversity converges upon a ‘common diplotypic proteome (CDP)’, a distinctive subset of genes preferentially encoding two different proteins. Moreover, we find that mutations predicted to alter protein function exist, in each of the 386 genomes, significantly more frequently in *cis* than in *trans*, with an average *cis*/*trans* ratio of 60:40. In addition, we observe different classes of *cis*- and *trans*-abundant genes. With these results, we identify key features characterizing the diploid landscape in human genomes (for overview see Fig. 1), and contribute novel insights into the ‘true nature of genetic variation’, which cannot be understood without knowing the distribution of variants on each of the two parental sets of chromosomes.

## Results

### Twelve molecular haplotype-resolved genomes

To haplotype-resolve 12 individual genomes from a representative German population cohort, we applied our fosmid pool-based next generation sequencing approach, described in detail earlier^4^,^12^ and corroborated by resolving HapMap trio child NA12878 (ref. 5) (Methods). Between 32 and 52 super-pools were sequenced for each of the 12 individuals on SOLiD platform. This generated between 20 and 63 Gb of uniquely mapped reads per genome, resulting in 3.5–12.5 × mean haploid genome coverage with, on average, 89% of the autosomes covered at >2 x. Depending on the number of pools sequenced and the read coverage obtained, up to 818,000 phase-informative fosmids (400,000 on average) were detected in each individual genome (Supplementary Table 1; Supplementary Fig. 1a,b). Fosmids were found to be roughly distributed equally between the two haplotypes of each autosome. The single nucleotide polymorphism (SNP) alleles in fosmid sequences and phase-informative heterozygous SNPs were called and validated as described^4^. Heterozygous SNP calls correlated with the number of pools sequenced and fosmids detected (Supplementary Fig. 1c,d). Rigorous SNP filtering was performed (Methods); false positive call rates, likely to reduce phasing accuracy, were 0.2% on average (0.08–0.50%); for additional accuracy estimates see Supplementary Table 2. In each of the individual genomes between 1.21 and 2.44 million SNPs were detected, corresponding number of phase-informative, heterozygous SNPs were between 0.48 and 1.41 million (Supplementary Table 3a). Notably, in the region of highest variability, major histocompatibility complex (MHC), up to 11,789 total SNPs and 7,841 heterozygous SNPs were identified. A detailed survey of heterozygous SNPs, their characteristics, distribution, functionally relevant classes and potential disease relevance is given in Supplementary Table 4.

We phased each of these 12 individual genomes by applying RefHap^13^ (Methods), resolving the vast majority of their detected SNPs (86–99%). The extent and completeness of phasing correlated with sequencing depth; 58–81% of the autosomes were assembled into ~8,000–16,000 contigs of up to 5.2 Mb, with an N50 contig length^6^ of 340 kb on average (range 169–629 kb) in the seven most completely resolved genomes (Supplementary Table 3a). In particular, the highly complex MHC region was phased across 75–97% of its length, with an average N50 length of 1.3 Mb (range 220 kb–3.1 Mb). The extent of phasing allowed determination of the concrete molecular haplotype pairs for up to 12,976 autosomal genes (73%) per genome, 48% on average (Supplementary Table 3b), including 10 kb upstream regions in nearly half of the cases. Between 598 and 1,630 potentially damaging mutations per individual (predicted by use of SIFT^14^ and PolyPhen-2 (ref. 15)) (Methods) and between 886 and 1,478 genome-wide association (GWA) SNPs (Supplementary Table 4) were assigned to phase context.

We then assessed the extent and nature of molecular diplotypes within each individual diploid genome. To which extent do maternal and paternal chromosomes encode different genes, proteins and potential regulatory sequences? Up to 84% of all autosomal genes (primary transcripts) contained at least one heterozygous SNP and so had two different molecular forms. The 10 kb upstream sequences were diplotypes in up to 78% of cases, and the transcripts together with upstream sequences produced diplotypic gene regions in up to 95% of cases. The vast majority of all diplotypes, ~90%, contained two or more heterozygous SNPs that could exist in either *cis* or *trans* configurations and therefore required phasing. We were able to determine the concrete pairs of molecular haplotypes in up to 95% of cases, 65% on average (Supplementary Table 5a). Consistently 16–22% of all genes within each individual diploid genome were found to encode two different proteins, defined by the presence of at least one non-synonymous SNP (nsSNP) causing an amino acid (AA) exchange. Between 3 and 6% contained two or more AA exchanges and 1% on average two or more potentially perturbing AA exchanges, the concrete *cis* or *trans* configurations of which we resolved in up to 86% of cases, 66% on average (Supplementary Table 5b). Between 57 and 73% of these mutations were found to reside in *cis* and between 27 and 43% in *trans*. Taken together, substantial and overall similar fractions of gene and protein diplotypes constitute the molecular foundation of organismal function within each of these individual genomes. The question is now, how many different, unique molecular diplotypes and underlying haplotypes do exist in this sample and to which extent these are shared.

### Immense diversity of haploid and diploid gene forms

The related, more general question concerns the entirety of unique haploid and diploid gene forms that constitute the ‘diploid hardware’ of cellular and organismal functions and their variation in population samples of defined size. Can saturation be reached when size increases? With MP1 (ref. 4) and NA12878 (ref. 5), a total set of 14 molecularly haplotype-resolved genomes provided the basis for population-level analyses. These genomes allowed comprehensive comparative evaluation of molecular versus statistical phasing data (Methods). With an overall phase discordance of 3.6% for exome data, 5.3% for transcript data and 5.9% genome-wide data (Supplementary Table 6a,b; Supplementary Note 1), the complementary use of 1000G statistical haplotype data appeared suitable to corroborate preliminary results, and should facilitate revealing larger patterns or differences, if present. To scale up analyses, we utilized 57CEU^16^ and the entire set of European ancestry-based genomes, 372EUR^11^.

**Figure 2:**
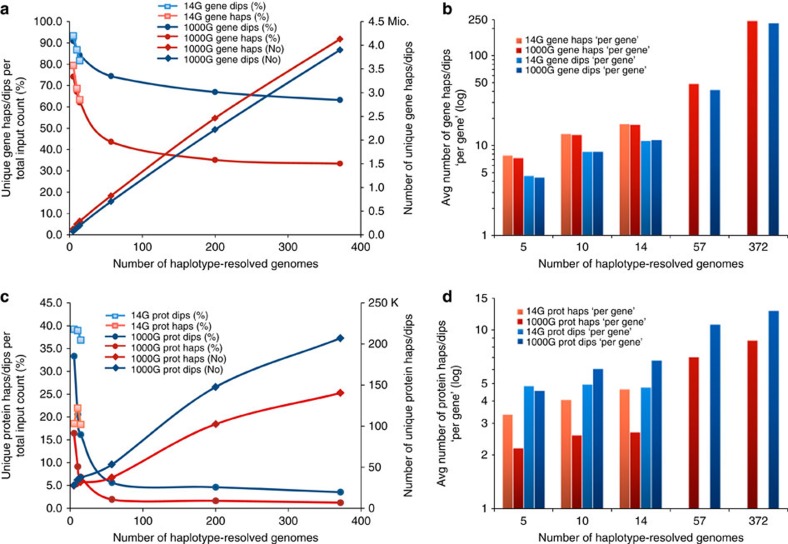
Diversity of unique gene and protein haplotypes and diplotypes. Overall scheme: the number of unique, different haplotypes (red colours) and pairs thereof, diplotypes (blue colours) presented relative to increasing number of haplotype-resolved genomes, drawn from the 14 molecularly resolved (14G) and statistically resolved genomes of European ancestry from the 1000G database^11^,^16^ (colour/symbol codes in inboxes); (**a**,**b**) gene haplotypes and diplotypes; (**c**,**d**) protein haplotypes and diplotypes; (**a**,**c**) haplotypes and diplotypes expressed as whole-genome counts and (**b**,**d**) as global averages ‘per gene’. (See also Supplementary Note 2). Haplotypes and diplotypes correspond to autosomal RefSeq Hg18 genes from UCSC table browser. (**a**) Decreasing curves present fractions of unique gene haplotypes and diplotypes relative to all measured haplotypes and diplotypes; increasing curves present their absolute numbers. (Note that total diplotype input count equals half of haplotype input count.) Data points correspond to 5, 10 and 14 genomes from 14G (squares as colour coded) and 5, 10, 14, 57, and 372 genomes of European ancestry derived from 1000G database (symbols, colour-code in inbox). An additional data point, 200 genomes (1000G) were integrated to anchor graphs. Haps, haplotypes, dips, diplotypes, No, number. (**b**) Unique gene haplotypes and diplotypes presented as global averages ‘per gene’ for given sample sizes; number of unique haplotypes and diplotypes were added up across given number of haplotype-resolved genomes and divided by the number of autosomal genes that were variable across given sets of genomes (>93%). (Note that the absolute number of diplotypes equals half of the haplotypes, explaining the relatively lower number of unique diplotypes). Data are shown for 14G and subsets thereof and 1000G-derived sets of genomes, as indicated by their colour-coding. Avg, average. (**c**) Unique protein haplotypes and diplotypes analogous to **a**. Prot, protein. (**d**) Average number of unique protein haplotypes and diplotypes ‘per gene’ analogous to **b**.

At first, we determined the entirety of unique haplotypes in relation to increasing number of genomes (Methods; Supplementary Note 2). Sets of 5, 10 and 14 molecularly haplotype-resolved genomes were examined and corresponding subsets were extracted from 57CEU. Selection bias was controlled and proved negligible (Supplementary Table 7; Supplementary Methods). In five molecularly phased genomes, the entirety of unique haplotypes amounted to 79.5% of all measured haplotypes (~60,000). The corresponding fraction obtained from 57CEU was 74.3%, at a full haplotype count of ~166,000; thus, ~36% of all haplotypes could be analyzed across these subjects at the molecular level. In 10 and 14 molecularly phased genomes, fractions of 68.7% and 63.4%, respectively, were unique and corresponding fractions in 57CEU were highly similar. The fraction of unique haplotypes decreased to 43.7% in 57CEU and reached with 33.5% the flat part of the curve in 372EUR (Fig. 2a; Supplementary Table 8a). To conclude, where we study small number of genomes such as five, there is roughly a 75% chance that any haplotype encoding a gene-of-interest may be unique; that is, not yet having been identified in any other genome. In 57 genomes, almost every second haplotype is expected to be unique and over a population size of 300, roughly every third.

The absolute number of unique haplotypes increased substantially (34-fold) from 5 to 372 genomes, with 4.1 million different gene forms still far from reaching a plateau (Fig. 2a; Supplementary Table 8b). Diversity corresponded to 8, 14 and 18 unique haplotypes ‘per gene’, an average across all autosomal genes and 5, 10 and 14 molecularly resolved genomes, respectively. The corresponding numbers extracted from 1000G were nearly identical, and increased to 50 and 249 haplotypes ‘per gene’ (Fig. 2b; Supplementary Table 8c). Finally, the diversity of haplotypes encoding potential regulatory, 10 kb upstream sequences was, with 2.8 haplotypes per kb on average, even larger than genic haplotype diversity (2.2 haplotypes per kb).

The analysis of both, haplotypes and diplotypes is equally important and biologically meaningful. Because differential expression is widespread^17^,^18^,^19^ either one of the haplotypes or the diplotype can exert ‘gene function’, potentially creating three different biological states. Both haplotypes and diplotypes can play a key role in disease^9^,^20^,^21^. An importance of diplotypes over haplotypes in relation to drug response has been demonstrated^22^. The diversity of diplotypes was even higher: their fractions relative to total diplotype count amounted to 93.4% in 5, to 81.8% in 14 genomes; corresponding results from 1000G were nearly identical. About 75% of all diplotypes were unique in 57CEU and over 60% in 372EUR (Fig. 2a; Supplementary Table 8a). Their absolute numbers exhibited an even stronger increase than haplotypes and almost reached an equal amount, 3.9 Mio, in 372EUR, indicating increased combinatorial space with growing abundance of molecular haplotypes (Fig. 2a; Supplementary Table 8b). Such diversity corresponded to a range of 5 to 236 unique diplotypes ‘per gene’ on average (Fig. 2b; Supplementary Table 8c). Evaluation of probable bias of haplotype/diplotype quantification introduced by phasing (switch) errors resulted in a potential overestimation of molecular haplotypes by ~14%, of statistical haplotypes by ~25% (Methods; Supplementary Methods). This would reduce the number of unique haplotypes to 3.1 Mio in 372EUR, lowering the fraction of unique gene haplotypes relative to total input count at most by ~8%.

Taken together, the analysis of multiple haplotype-resolved genomes unveiled an exorbitant diversity of both haploid and diploid gene forms, encoding potential variation in gene function in the European population. Providing a first quantitative framework, our data allowed extrapolation of the number of unique ‘haps and dips’ for much larger European ancestry-based samples. For example, between 1.7 and 2.3 billion unique haplotypes and between 4.3 and 5.5 billion diplotypes were projected for one million genomes (Methods; Supplementary Table 9), the lower numbers were corrected for potential overestimation.

### No major gene form in the vast majority of genes

With an immense diversity of gene forms evident, we moved to the single gene level, asking to what extent our results were compatible with a conventional conception of a ‘wild-type’ or major gene form. We classified all genes by frequency of occurrence of their haplotypes and diplotypes into three distinct categories: (1) Genes that have one major haploid or diploid form, which accounts for at least 50% of the measured haplotypes or diplotypes; (2) Genes that have at least one common haplotype or diplotype, defined by a frequency of ≥20% and (3) Genes that exhibit only haplotypes or diplotypes below this frequency threshold.

**Figure 3:**
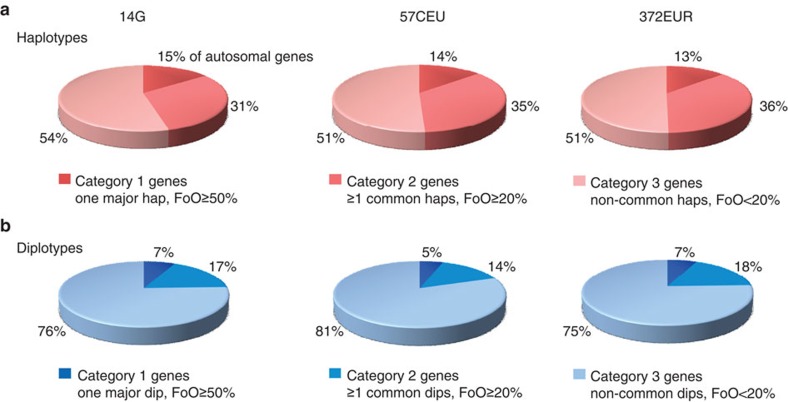
Categorization of autosomal genes. Pie charts show classification of autosomal genes into three categories based on frequency of occurrence (FoO) of their unique haplotypes (red colours) and diplotypes (blue colours). For instance, category 1 includes all genes that have one predominant haplotype or diplotype, accounting for ≥50% of all measured haplotypes or diplotypes; the definitions for categories 2 and 3 genes are analogous. Fractions of these categories (%) are relative to the total of autosomal RefSeq Hg18 genes assigned. Data shown for the sets of 14 molecularly haplotype-resolved genomes (14G) and 57CEU and 372EUR statistically resolved genomes derived from 1000G database^11^,^16^.

Strikingly, genes that had one major haplotype (category 1) represented by far the smallest fraction of all autosomal genes. With 14–15% nearly identical in 14 molecularly resolved genomes, 57CEU and subsets thereof, the fractions remained in the same range even in much higher number of genomes (372EUR) (Fig. 3a; Supplementary Table 10a). This category included 2,242 genes on average, their major gene forms accounting for ~70% of all haplotypes independent of sample size (Methods; Supplementary Table 10b). This set of genes was significantly enriched^23^ for G-protein coupled receptor genes (GPCRs), genes involved in immune functions and developmental genes including those involved in brain development (*P*-values between 2.61 × 10^−70^ and 9.73 × 10^−04^; Methods). The average number of haplotypes ‘per gene’ was between 3 and 22, much lower than the global averages described above (Supplementary Table 10c). These results demonstrate that the vast majority of genes, over 85%, do not encode one predominant haplotype. ‘Therefore, the concept that there is one predominant or ‘wild-type’ form of a gene and few rare or ‘mutant’ forms is overly simplistic and misleading’ (quote from the study by Stephens *et al*.^24^).

The rule rather than the exception is that each gene represents the equivalent of multiple different forms, which account for only limited fractions of all haplotypes observed. Category 2 genes that have at least one common haplotype with a frequency ≥20% were found to constitute roughly one third of all genes (Fig. 3a; Supplementary Table 10a). Notably, all common haplotypes accounted for less than half (43–47%) of all haplotypes (Supplementary Table 10b). Category 2 genes encoded mainly immune functions (other than category 1) and translational mechanisms (*P*≤1.79 × 10^−6^–0.0013). Category 3 comprised the majority of all autosomal genes, with over half constituted by non-common haplotypes with frequencies <20% (Fig. 3a), substantial fractions of which were ‘singleton haplotypes’ (Supplementary Table 10b). Such fragmentation of gene forms was underscored by the fact that much higher number of haplotypes ‘per gene’ (up to 399 on average) were determined (Supplementary Table 10c). Category 3 was strongly enriched for genes that play an important role in regulation of the functions of the nervous system and behaviour (*P*≤5.35 × 10^−16^–4.04 × 10^−5^) (Supplementary Note 3). As expected, the haplotype spectra of genes correlated with their number of SNPs and lengths (Supplementary Fig. 2a–h). The lists of genes in each of the three categories are provided (http://www.molgen.mpg.de/~genetic-variation/genes_categories/).

Classifying genes by their diplotype spectra unveiled an even higher complexity constituting diploid gene function. Once any two haplotypes of a gene combine to make a diplotype, its resulting frequency is always lower than the frequency of either of the parental haplotypes. Thus, the fractions of genes that have one major diplotype were reduced to about half (5–7%) across all sample sizes (Fig. 3b; Supplementary Table 10a–c). The same applied to the category of genes that have at least one common diplotype (14–18%). As a consequence, the fractions of genes that exhibited non-common diplotypes were found largely expanded (75–81%); importantly, as many as one half of all genes in this category were encoded by diplotypes with frequencies <5% or ‘singleton diplotypes’.

### Protein haplotype and diplotype diversity

How does this diversity of gene forms translate into diversity of protein forms? Analyses were performed analogous to those described above, using the subset of nsSNPs that cause AA exchanges. With 1.3–1.7 nsSNPs on average in the variable genes and considerable fractions of invariable coding regions, the diversity of haplotypes and diplotypes at the protein level was substantially reduced. The entirety of unique protein haplotypes relative to all measured haplotypes accounted for 18.5% in five genomes; similar results were obtained from the corresponding 57CEU-derived subset. The fractions decreased to 2% in 57CEU and 1.1% in 372EUR (Fig. 2c; Supplementary Table 11a). The entirety of unique protein diplotypes amounted to over 33%, 5.6% and 3.4% of total diplotype count in 5, 57 and 372 genomes, respectively. These fractions were equivalent to ~28,000, 53,000 and 207,000 different protein diplotypes encoding potential variation in protein function (Fig. 2c; Supplementary Table 11b).

Translating these whole-genome-based estimates into global averages ‘per variable gene’, roughly 2–9 unique protein haplotypes and 5–13 unique protein diplotypes were calculated for 5 up to 372 genomes (Methods; Fig. 2d; Supplementary Table 11c). For extrapolations up to one million genomes see Supplementary Table 9. Over 80% of the variable genes had one major protein haplotype, over 63% had one major protein diplotype and one third had common protein diplotypes (5–20 on average). A small fraction, roughly 4%, consisted of non-common and private protein diplotypes only (Supplementary Table 12). For ‘personal protein diplotype signatures’ see Supplementary Table 13.

Taken together, the diversity of haploid and diploid protein forms was much lower than their counterparts at the DNA sequence level. The absolute numbers were, however, still considerable, particularly of the protein diplotypes, up to 90% of which encoded two different proteins in the population. These can allow huge functional versatility of diploid genomes as an inherent key feature of diploidy and play an important role in biological variation within and between cells and organisms.

### A common diplotypic proteome

To further dissect the potential functional importance of diploidy, we asked whether certain classes of genes were particularly likely to encode diplotypic proteins. Across all genomes, consistently between 16 and 22% of the autosomal genes (18% on average) were found to encode two different proteins (Supplementary Tables 5b and 14). Thus, to what extent do these genomes share genes that occur as protein diplotypes; is there a subset of genes that encode two different proteins particularly frequently? In other words: do mutations ‘rain’ over all genes, or preferentially affect specific classes of genes? So we analyzed, first, the distribution of diplotype frequencies for all genes in each of the four samples: our 14 molecularly phased genomes and a corresponding set selected from 57CEU, the entire set of 57CEU and 372EUR. Second, we extracted, from each of these samples, sets of genes that were diplotypes in increasingly larger fractions of genomes. Third, we tested to which extent the extracted sets of genes overlapped to derive a common subset of genes that preferentially encode two different proteins in European population samples, which we termed as ‘common diplotypic proteome’.

**Figure 4:**
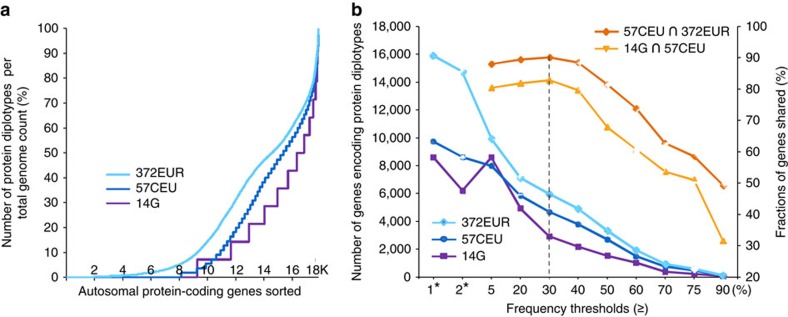
Subsets of autosomal genes encoding protein diplotypes. (**a**) Autosomal genes sorted by increasing protein diplotype frequencies in the sets of 14 molecularly haplotype-resolved (14G), 57 and 372 statistically resolved genomes from 1000G database^11^,^16^, as colour-coded. The *x*-axis indicates the number of (sorted) genes. Diplotype frequency of a gene defined by its number of protein diplotypes relative to the total number of genomes examined (*y*-axis). Protein diplotype defined by presence of at least one nsSNP. (**b**) Number of autosomal genes encoding protein diplotypes as a function of increasing frequency thresholds, 5 to 90% of total genome count (*x*-axis). For example, roughly 3,000 genes exhibit protein diplotypes in at least 30% of the 14 molecularly resolved genomes (14G) and roughly 6,000 genes in at least 30% of 372EUR genomes (colour-coded). ‘*’ indicates the number of genes encoding protein diplotypes in at least one or two genomes in each of the three sample sets shown. The two graphs in the upper part show, for each of the frequency thresholds, the percentages of genes encoding protein diplotypes that are shared (*y*-axis right) when 14G and 57CEU-derived gene sets (yellow graph) and 57CEU and 372EUR-derived gene sets (orange graph) were intersected (∩). Percentage values of genes refer to the smaller sample set. The dotted line marks the frequency threshold resulting in the highest overlap. The number of genes exhibiting diplotype frequencies above this threshold of 30%, 5,951 in 372EUR and 4,665 in 57CEU, were significantly higher as compared with chance (*P*<4.6 × 10^−9^ and *P*<9.3 × 10^−3^, respectively) based on a binomial test.

To begin with, we counted for each of the 17,861 autosomal protein-coding genes the number of genomes where the gene existed as a protein diplotype (Methods). ‘Diplotype’ was scored by presence of any one or more of nsSNPs, essentially considering it a property of the gene. Then we sorted the genes by increasing diplotype frequencies. In all data sets, the sorting showed a highly similar distribution of diplotype frequencies, with increasingly smaller number of genes being diplotypic in increasingly higher number of genomes. At the extreme, genes were diplotypic in all genomes (Supplementary Fig. 3a–d). Plotting diplotype frequencies relative to total genome count (100%), the curves were highly parallel at frequencies >5% (Fig. 4a). We extracted, from each of the samples, the concrete gene sets that exhibited protein diplotypes with frequencies above defined thresholds, from 5 up to 90% of total genome count. Gene numbers were roughly in the same range for defined frequency thresholds, showing an overall parallel decrease (Fig. 4b; Supplementary Table 15a).

We then examined to what extent these extracted gene sets were the same. Thus, we analyzed the overlaps between our 14 molecularly resolved and 57CEU genomes, and between 57CEU and 372EUR, separately for each of the defined frequency thresholds (Methods). The strongest overlaps were found at a frequency threshold of ≥30% (Fig. 4b; Supplementary Table 15b). The number of genes exhibiting diplotype frequencies above this threshold, 5,951 in 372EUR and 4,665 in 57CEU (Supplementary Table 16a), were significantly higher as compared with chance (*P*<4.6 × 10^−9^ and *P*<9.3 × 10^−3^, respectively, based on a binomial argument) (Supplementary Methods). Integrating the sets of genes contained within the overlaps (Methods), we obtained a total of 4,269 genes that were shared by at least two distinct sample sets, the ‘CDP’. These genes also shared 88% with an expanded set of 628 phased genomes from the 1000G database^11^. A subset of the CDP, 793 genes, contained two or more potentially functionally significant mutations, which can reside in either *cis* or *trans* configurations. Thus, this gene set represents a common core set of ‘phase-sensitive’ genes in the European population, where the phase of mutations is particularly likely to be of critical importance for protein function, phenotype and clinical genome interpretation. These gene sets are available for download at http://www.molgen.mpg.de/~genetic-variation/common_diplotypic_proteome/.

The CDP showed a significant overrepresentation of certain gene ontology (GO) groups (global tests *P*<0.001–0.009), using the programme FUNC^23^ (Methods). These groups included GPCRs, in particular olfactory receptors (ORs), and other membrane and cell-surface proteins, as well as proteins related to the immune system, such as the MHC (Class I and II), and drug metabolism (*P*<2.36 × 10^−40^–0.003). These results were corroborated by the analysis of functional pathways using the ConsensusPathDB^25^ (Methods); gene sets showed highly significant overlaps (*P*<7.66 × 10^−39^–2.38 × 10^−05^) with pathways involving OR and GPCR-related signalling and signal transduction, (extra-cellular matrix related) processes of inter-cellular communication, immunoregulatory processes and membrane-linked drug transport. Importantly, genes involved in Alzheimer’s disease and various kinds of cancers were found to be highly and significantly enriched (*P*<5.4 × 10^−15^). The potential significance for cancer of the CDP was supported by strong overlaps with genes contained in the COSMIC cancer database (*P*<1.89 × 10^−14^–2.73 × 10^−4^). In addition, the common core set of ‘phase-sensitive’ genes was found strongly enriched for a spectrum of immune diseases and diabetes (*P*≤2.6 × 10^−6^–6.05 × 10^−5^). Moreover, we uncovered a strong overrepresentation of transcription factors (TFs) (hyper-geometric test, *P*<0.02), especially Krüppel-type zinc finger TFs (hyper-geometric test, *P*<1 × 10^−6^). Taken together, the extraction of a CDP allows focusing the potential functional impact of diploidy on definable classes of genes. These primarily play a role in inter- and intra-cellular signalling and immune processes, presumably to modulate cell–cell communications and fine tune expression patterns in cells.

### *Cis*- versus *trans*-abundant genes

**Figure 5:**
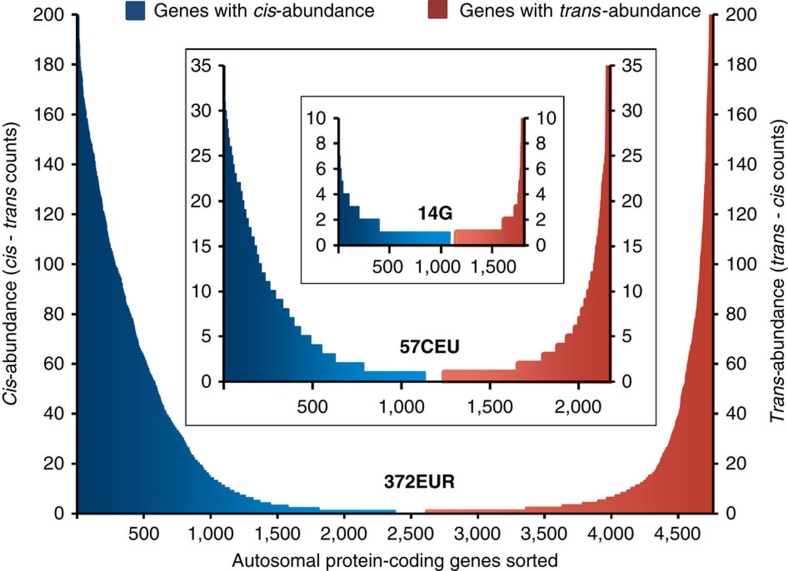
*Cis*- versus *trans*-abundant genes. Autosomal protein-coding genes sorted by decreasing *cis*-abundance from left to right. The *x*-axis indicates the number of (sorted) genes. *Cis*-abundant genes (blue) defined by positive differences between their *cis* and *trans* counts (*y*-axis left). For *trans*-abundant genes (red), initially calculated as increasingly negative differences and sorted towards the right end of the *x*-axis, the absolute differences are presented as *trans–cis* counts (*y*-axis right). Curves are shown for the sets of 372EUR and 57CEU statistically haplotype-resolved genomes from 1000G database^11^,^16^ and for the 14 molecularly resolved genomes. GO groups with a preference for either end of the spectrum are described in text.

To analyze protein diplotypes in more detail, we examined the distribution of mutations on each of the two parental chromosomes. Are mutations distributed randomly, or can we distinguish patterns of phase? We assessed all autosomal protein-coding genes with two or more potentially perturbing mutations, counting the number of *cis* and *trans* configurations for each gene in each of the sample sets described (Methods). We then determined for each gene the difference between *cis* and *trans* counts as a measure of abundance of either configuration. Sorting the genes by difference revealed, from left to right, ‘*cis*-abundant’ genes with very positive (>0) values declining to very negative (<0) values, indicating ‘*trans*-abundant’ genes. Expressing the negative values as absolute differences resulted, in all samples, in U-shaped curves with exceedingly *cis*- or *trans*-abundant genes at the extreme ends and a relatively long corridor of ‘mixed’ genes in between (Fig. 5). Overall, *cis* abundance was more pronounced, as indicated by a mean difference of 153 for *cis*-abundant genes, compared with 113 for *trans*-abundant genes. Subsequently we composed a superset of all *cis* and *trans* configurations, providing the basis for further analyses (Methods).

To examine whether certain groups of genes encoding protein diplotypes tended to exist preferentially in *cis* or *trans* configurations, we performed a GO enrichment analysis using the rank-test option of FUNC. This allows identification of GO groups with a preference for either end of the spectrum without setting a concrete cutoff criterion. While the global test was significant for all three taxonomies for genes with *trans*-abundance (*P*<0.002–0.025), only ‘molecular function’ was significant for *cis* abundance (*P*=0.007), indicating that genes with *trans*-abundance are more strongly enriched for certain functional groups. In particular, immune genes, membrane-related genes, genes related to drug response, cell metabolism and fate dominated among the *trans*-abundant genes (*P*<8.25 × 10^−09^–0.0001). The ‘molecular functions’ enriched among *cis*-abundant genes were GPCRs, particularly ORs, and extra- and intra-cellular signalling molecules (*P*<1.55 × 10^−25^–4.03 × 10^−09^). Thus, we were able to further differentiate some of the GO groups found enriched in the CDP with respect to their preferential configurational profile.

Furthermore, *cis*- and *trans*-abundant genes were found distinctively enriched for a number of pathways: *cis*-abundant genes for ‘signal transduction’ (*P*<6.69 × 10^−13^), *trans*-abundant genes for (MHC related) immune responses, inter-cellular immunoregulatory interactions and autoimmune processes (*P*<3.06 × 10^−05^). Finally, *trans*-abundant genes were found strongly overrepresented in gene sets associated with a broad spectrum of immune and autoimmune system diseases, diabetes mellitus, asthma and different types of cancers (*P*<1 × 10^−5^), while *cis*-abundant genes were underrepresented in disease gene sets with the exception of ‘tumour thrombi’ (*P*=0.048). These results indicate that certain sets of genes preferentially occur in *cis* or *trans* configurations, that pathways may distinctively be influenced by either *cis*- or *trans*-abundant genes, and that *trans* configurations are more often associated with diseases.

### Whole-genome *cis* abundance of mutations 60:40

Overall, *cis* configurations, leaving one form of the gene unperturbed, would be expected to occur more frequently in an individual genome to preserve organismal function. Thus, we determined the ratio of *cis* to *trans* configurations across all autosomal protein-coding genes for each individual genome in all sample sets (Methods). In fact, a striking phase imbalance was observed: without any exception, in each of the 14 molecularly phased genomes (Supplementary Table 16), 57CEU (Supplementary Table 14) and 372EUR statistically phased genomes, *cis* configurations of potentially perturbing mutations occurred significantly more frequently than *trans* configurations (*P*<7.68 × 10^−8^–2.11 × 10^−3^), resulting in an overall ratio of about 60:40 (Table 1; Supplementary Note 4). This global *cis* abundance with a ratio of 60:40 was also observed, when expanding analyses to the total set of nsSNPs (*P*<5.98 × 10^−7^–1.33 × 10^−5^; Table 1). We dissected *cis* and *trans* configurations further in relation to the number of mutations contained within a gene. This allowed at the same time controlling for a potential overestimation of *cis* configurations due to lower coverage of some genomes. By far the most frequent configurations, >70%, were pairs of mutations, the majority (67%) of which resided in *cis* (Supplementary Table 17a). The second most frequent configurations (16–18%) were combinations of three mutations, occurring in equal proportions in *cis* or *trans*. As expected, with growing number of SNPs, *trans* configurations were found to dominate increasingly, for example, 70:30 in the case of five SNPs. Again, the results from both molecularly and 1000G-resolved genomes were nearly identical. The same applied to the configurations constituted by nsSNPs (Supplementary Table 17b).

Global *cis* abundance is mainly driven by pairs of mutations that are overwhelmingly in *cis*. Dissecting this result further, we examined the *cis*/*trans* ratio in relation to inter-mutation distance. For average distances between 20 and 27,446 bp, the *cis* fractions were between 82 and 62%. The remaining 10% of mutation pairs were in *cis* in at least 50% of cases, up to a distance of 93,765 bp (Supplementary Fig. 4a–c; Supplementary Note 5). To examine the *cis*/*trans* ratio in relation to mutation frequency, we compared pairs of common mutations (average frequency 0.23) with pairs of rare mutations (average frequency 0.0037), resulting in *cis* fractions of 84.3% and 49.4%, respectively (Supplementary Note 6).

### Differences in phase configurations

As shown by Benzer^2^ and others^26^,^27^, *cis* versus *trans* configurations between identical (null) mutations, even Mb apart, can result in profound alterations of phenotype and disease progression. Therefore, pairs of identical mutations that can ‘switch’ phase may be particularly important. Performing all possible pair-wise genome comparisons (69,192) across the largest available set of 372EUR allowed extraction of a set of 1,047 ‘phase-alternate’ genes (http://www.molgen.mpg.de/~genetic-variation/phase_alternate_genes/; Methods), which carry pairs of identical, potentially perturbing mutations in both *cis* and *trans* configurations. Although identical in mutational genotype, these genes can have different underlying haplotypes. Thus, the clinical and functional interpretation of mutations in these genes may critically depend on their phase in any individual genome. This set of genes was found significantly enriched for a variety of diseases including Alzheimer's disease, immune system diseases, different types of cancers and diabetes mellitus (*P*<7.49 × 10^−7^–0.0000).

In a second step, we expanded our analyses of pairs of potentially perturbing mutations to larger distances in the Megabase range (Methods). In principle, differences in phase configurations between any two diploid genomes were evaluated at adjacent, shared heterozygous positions along phased sequences, using a ‘sliding window’ approach. Performing 69,192 pair-wise genome comparisons allowed identification of all pairs of mutations that can ‘switch’ phase in any genome of the sample (372EUR; Methods). The results are presented as a sorted, genome-wide list of 23,801 ‘anchor’ mutations shown in conjunction with their 3′ downstream ‘phase-alternates’ (http://www.molgen.mpg.de/~genetic-variation/phase_alternate_mutations/). This resource may assist interpretation of potentially functionally and phenotypically relevant mutations in the context of phase in European ancestry-based samples.

## Discussion

To gain further insight into the ‘fundamental importance of diploidy’^28^ for the understanding of human genomes and disease, we have performed a population-level analysis of nearly 400 haplotype-resolved genomes of European ancestry. We have extracted key features characterizing the diplotypic nature of human genomes: an immense diversity of both gene haplotypes and diplotypes underlying variation in gene function; a common diplotypic proteome and non-random patterns of *cis* and *trans* configurations of mutations, with an ‘unfailing’ global 60:40 *cis* abundance and distinguishable classes of *cis*- versus *trans*-abundant genes. With our results, we provide a conceptual, analytical and quantitative framework charting the yet largely unexplored diploid landscape. Moreover, we provide rich resources including data sets of common diplotypic genes/proteins, which will facilitate targeted approaches at all omics levels to explore the role of diploidy for cellular, organismal and phenotypic diversity, within and between species, in health and disease. Finally, our portrait and quantification of the ‘true’ molecular basis underlying individual variation in gene and genome function highlights both the indispensability and challenges of diplotype analysis for the development of valid approaches to precision medicine.

In our work, we have integrated sets of molecularly and 1000G statistically haplotype-resolved genomes^11^. At an initial discordance of few per cent, the smallest between exome data (4%), both data sets yielded remarkably similar results. This shows that 1000G data are suitable to address key questions concerning the nature of (functional) diplotype architecture in a population, and generate hypotheses towards potential functional implications of diploidy. Where, however, personal genome analysis calls for maximum phasing accuracy to interpret clinically relevant variation validly, deep molecular haplotyping is required.

Our global view of haplotype/diplotype diversity in relation to population size suggests that current efforts are still far from capturing the majority of gene forms and that saturation may not even be achievable. The concept of a predominant, ‘wild-type’ form of ‘the’ gene appears obsolete for over 85% of genes, challenging traditional ‘Mendelian’ views. This highlights the need for an expansion of current concepts of ‘the’ gene^29^, along with the development of appropriate documentation and language. The enormous diversity of haploid and diploid gene forms raises fundamental questions concerning the relationships between sequence(s), structure(s) and function(s)^21^. Computational approaches are required to condense complexity, for instance classification of functionally related/similar sequences^20^. Furthermore, experimental approaches are required, which focus firstly on the analysis of combinations of variants as compared with single mutations^22^, and secondly three different states per gene, either one of the haplotypes and the diplotype. Finally, our estimates of diversity represent yet the lower end, anticipating the challenges of incorporating structural variation^30^ and increasing the number of genomes.

Exorbitant diversity at the gene level was found to converge upon a common diplotypic proteome, a subset of genes preferentially encoding two different proteins, allowing gene functions to be differentially exerted and/or diversified. Thus, the CDP may represent a ‘major modulating principle’ generating diverse cellular and physiological outcomes in individual organisms, and at the population level giving rise to phenotypic diversity, adaptive and evolutionary processes^1^,^4^. Enrichment results suggest an important role of diploidy for preserving flexibility of receptor-mediated cell–cell communications, immune-related processes and transcriptional regulation. The ‘wrong’ alleles being active, the CDP may prepare the ground for pathological conditions, as was supported by strong enrichment of cancers, Alzheimer’s, immune and other diseases. Strikingly, several diplotypic gene classes such as ORs and other cell-surface/receptor or immune-related proteins are known to be subjected to mono-allelic expression^18^,^31^. Thus, the relationship between the CDP, mono-allelic expression^32^ and also allele-specific expression^17^,^19^ requires clarification. So does the role of the CDP in transcriptome diversity within and between individuals, species, tissues, developmental and physiological stages, health and disease. The CDP represents a new aspect of the highly complex proteome^33^, as such subject to further diversification through alternative splicing and post-translational modifications. The molecular validation of the mutations defining these protein diplotypes will present future challenges^34^.

The extent and nature of diplotypes in individual genomes and the population as a whole demonstrate the importance of phase-sensitive approaches for precision medicine. Our data provide key information on the diversity of any potential target molecule for a drug candidate in the European population, suggesting models of population stratification for roughly 96% of all autosomal genes and ~5–20 different protein haplotypes/diplotypes per target. They enable moreover valid population-specific *in vitro* drug screening assays. Where optimization of individual treatment measures is, however, based on systems approaches, the existing protein alternates can introduce huge diversity of gene–gene interactions and functional outcomes. Thus, tailoring therapeutic measures to each individual will strongly benefit from further advances in this emerging field of diploid genomics.

## Methods

### Selection and characterization of individuals

The 12 individuals, MP2–13, were, as was MP1 (ref. 4), probands from the representative German population cohort PopGen^35^. At the time of ascertainment by random sampling, these individuals (three males and nine females) were between 53 and 60 years of age, without pathological findings in the physical examination or routine laboratory check (exclusion criteria), and without a history of severe diseases^4^. They were part of a ‘Haploid Reference Resource’ of 100 fosmid libraries generated from 100 probands and integral part of the ‘Max Planck Haplome Resource’. For all probands, genomic four-digit HLA-typing data were obtained; individuals carrying known MHC sequence haplotypes as well as unknown and disease-related haplotypes were selected. Genotypic data, generated by Affymetrix 1000 K typing were available for 11 individuals. All probands were of European ancestry and showed a strong correlation of >0.95 (linkage disequilibrium (LD) values) with the HapMap-CEU samples^4^,^35^. Ethical consent has been obtained from the Ethics Committee, the University of Kiel, and informed consent was obtained from the study participants.

### Fosmid library construction

High molecular weight DNA (genomic DNA (gDNA)) was prepared from 5 ml EDTA blood. Fosmid library preparation was carried out as described^4^; for necessary key steps see http://genome.cshlp.org/content/21/10/1672.full, for description in full detail see http://genome.cshlp.org/content/suppl/2011/08/03/gr.125047.111.DC1/Hoehe_GR_Supplementary.pdf. Briefly, 20 μg of gDNA were mechanically sheared to generate DNA fragments of ~40 kb. These fragments were used to prepare a complex fosmid library using the Epicentre EpiFOS Fosmid Library Production Kit according to the manufacturer’s protocol. The haploid ligation products were packaged into phage particles and amplified by transfection of *Escherichia coli* (Epi100) cells. Mass transfection generated ~1.44 × 10^6^ fosmid clones, equivalent to ~7 × coverage of each haploid genome. Mass transfected *E. coli* cells were distributed into three 96-well plates. Thus, each of the 288 wells contained random mixtures of ~5,000 fosmids, representing ~5% of the genome. These 288 pools were stored as glycerol-bacterial stocks to ensure long-term availability of the library. Complexity and evenness of genome representation were validated, as was library quality. To increase throughput, the three 96-well plates were combined into one, generating ‘super-pools’ of ~15,000 fosmids. The probability that complementary haplotypes may co-occur within a super-pool of ~15,000 fosmids is *P*<0.0112, which was verified by analysis of our fosmid pool-based sequencing data.

### Fosmid pool-based NGS to haplotype-resolve whole genomes

Multiple super-pools from these libraries were sequenced using a SOLiD platform, as described in detail^4^, see http://genome.cshlp.org/content/21/10/1672.full and http://genome.cshlp.org/content/suppl/2011/08/03/gr.125047.111.DC1/Hoehe_GR_Supplementary.pdf. Briefly, super-pools were bar-coded and up to 16 multiplexed pools were sequenced in a single flow cell. SOLiD sequencing libraries were prepared for 32 up to 52 unique fosmid super-pools per individual (Supplementary Table 1). With each pool covering ~15% of the genome, the sequencing of 32 pools from an individual’s library, each at 1–2 × coverage, was expected (based on earlier simulation studies) to result in a ~7–14 × whole-genome haploid coverage^4^. In total, 460 bar-coded fragment libraries and 8 mate-paired libraries were generated from purified fosmid pool DNA. Specifically, ~3 μg of purified fosmid DNA was sheared and size-selected DNA (100–150 bp) was ligated to SOLiD adaptor sequences containing unique barcode identifiers. Bar-coded SOLiD sequencing libraries were subjected to emulsion PCR. The quality of generated templated beads was checked by a work flow analysis (WFA) run prior to full sequencing of at most 650 million templated beads per slide (SOLiD V3+/V4). Mate-pair libraries were generated by circularization of sheared and size-selected fosmid DNA (insert size of 3.5 kb) and processed as described. In addition, a SOLiD sequencing library was prepared from pooled gDNA samples from 18 PopGen individuals and sequenced at lower coverage according to standard protocols to increase the accuracy of heterozygous SNP calling in low coverage regions.

### Fosmid detection

SOLiD reads were mapped and aligned to the reference genome (hg18) with Bioscope 1.3 (www.solidsoftwaretools.com) using default parameters. Duplicate reads, reads that did not map uniquely, and low quality reads (<q30) were removed. Approximately 80% of all sequencing reads were unambiguously assigned to unique barcodes, allowing separation of multiplexed sequencing reads into their original fosmid pool.

The detection of fosmid sequences was carried out as described^4^. Essentially, we examined coverage pile-ups of mapped reads in each sequenced fosmid pool. To begin with, the expected coverage was calculated as the ratio of total mapped bases to expected amount of DNA in the pool. The genomes were divided into bins of 1 kb, and each mapped read assigned to its appropriate bin. To detect the fosmids, we used a sliding window approach to locate suitable length regions containing reads above the coverage threshold defined dynamically based on the total number of mapped bases. Fosmids were identified as un-gapped contigs of 3 to 45 kb in length, with their chromosomal coordinates assigned (http://genome.cshlp.org/content/suppl/2011/08/03/gr.125047.111.DC1/Hoehe_GR_Supplementary.pdf).

### SNP detection and quality control

SNPs were called using the fosmid-aware SNVQ SNP caller, a novel Bayesian model for SNV discovery and genotyping based on quality scores^5^. To begin with, we verified for each fosmid pool that only one of the two alleles was present. Then, the fosmid sequences, which were separately obtained from each pool, were combined to one virtual pool to identify phase-informative heterozygous positions. We then performed fosmid-specific allele calls for these heterozygous positions using the SNVQ SNP caller. To generate a highly accurate input set for phasing, the following filters were applied: (i) SNPs with SNVQ quality scores <30 were removed; (ii) SNPs detected in regions, where the read coverage was too high (>50 × ) or too low (<3 × ), were removed and therefore variant loci with too low a coverage excluded from downstream analysis; (iii) novel heterozygous SNPs (not in dbSNP134) were required to have ≥3 × coverage of each allele; (iv) artificial SNPs generated by mapping the hg18 genome back to itself were removed; (v) SNPs overlapping with, or residing within, 3 bp of indels (dbSNP134) were removed. Thus, to eliminate false positives to ensure the accuracy of phasing, we have accepted a higher proportion of false negatives. Heterozygous and homozygous SNPs from Affymetrix 1000 K chip data were integrated into the SNP data set. For each individual genome, error rates for SNP calling were estimated using Affymetrix 1000 K data as reference (Supplementary Table 2).

### Fosmid pool-based phasing

Fosmid sequences were separated and tiled into contiguous molecular haplotype sequences based on allelic identity at multiple heterozygous positions within the regions of overlap. To this end, we applied our heuristic phasing algorithm ReFHap^13^, which can deliver highly accurate results even at low coverage and produce high-quality haplotypes at higher efficiency^13^. Briefly, the input matrix for ReFHap contains all haplotype-informative, heterozygous SNP positions, corresponding fosmid alleles and fosmid coordinates. The ReFHap phasing algorithm uses a graph construction that allows reduction of the SIH problem to the Max-Cut problem, facilitating highly precise assembly of fosmids (http://genome.cshlp.org/content/suppl/2011/08/03/gr.125047.111.DC1/Hoehe_GR_Supplementary.pdf). The quality of phasing was validated for each individual genome by comparison to 1000G phasing data. To assist phasing of the genetically highly complex MHC region, we integrated fosmid pool-based enrichment data, obtained from 11 individuals by using the Agilent SureSelect and NimbleGen systems.

### Biological analysis methods (I)

For analysis of autosomal genes, RefSeq genes^36^ were downloaded from UCSC table browser (hg18). All transcripts belonging to a gene were merged and the coordinates defining this entire region were used for subsequent analysis, resulting in a final set of 17,861 genes^4^. To predict a potential impact of an AA substitution on the structure and function of a human protein, we used PolyPhen-2 (ref. 15) and SIFT^14^. To optimize for sensitivity in this genome-wide analysis and detect all AAs that potentially affect an individual, we took the union of PolyPhen-2 and SIFT, using default threshold values of 0.85 and 0.05, respectively. The annotation of disease-related SNPs was performed using GWA Studies, the Genetic Association Database (GAD) and OMIM data obtained from UCSC (hg18) table browser.

### Use of 1000G consortium data

In the first phase of analyses, we utilized the then available Pilot Phase data set from 57 European ancestry-based samples, 57CEU^16^; haplotype data were downloaded from ftp://ftp.1000genomes.ebi.ac.uk/vol1/ftp/release/2009_04/). To expand our analyses, we used the Phase I data of 1,092 genomes^11^ (ftp.1000genomes.ebi.ac.uk/vol1/ftp/release/20100804/). We extracted the subgroup of 372 European ancestry-based samples (372EUR) including 57CEU; for details see Abecasis *et al*.^11^ Phased data were available across all genomes and genes, with ‘no call’ rates between 2.1 and 6% and routine use of imputation in the case of missing data^11^. Key results derived from Pilot Phase 57CEU data set were tested against Phase I 57CEU data ensuring validity of results. Key results were moreover corroborated in a total of 628 genomes including 256 genomes of East Asian origin^11^. 1000G annotation information (ftp.1000genomes.ebi. ac.uk/vol1/ftp/release/ 20100804/) was used to analyze protein diplotypes and the phase configurations of potentially damaging mutations in 57CEU and 372EUR.

### Molecular versus statistical phasing

For comparative evaluation, we phased the 12 molecularly resolved genomes statistically using the 57CEU data sets as the required supplementary population data source (ftp://ftp.1000genomes.ebi.ac.uk/vol1/ftp/release/2009_04/). We applied the programme fastPhase^37^ with default parameters and option p in order to utilize the 57CEU haplotype data sets to supplement the input data from each of the molecularly resolved genomes. The latter included the heterozygous, reference and non-reference calls that had been obtained for each genome from its combined fosmid sequence read data^4^, filtered for the SNP positions that the genome in question shared with the 57CEU data set. Subsequently, we compared molecular and statistical phase at adjacent SNP pairs using a ‘sliding window’ approach, and counted the number of phase-discordant SNP positions. In addition, phase discordance was evaluated for genes (primary transcripts) and exonic sequences. Phase discordance was calculated, dividing the number of phase-discordant SNPs assessed genome wide by the total number of heterozygous positions evaluated. The fractions of phase-discordant SNPs were also calculated separately for each of the 22 autosomal chromosomes and then averaged (results shown). The fractions of phase-discordant SNPs in transcripts and exonic sequences were assessed analogously. Finally, phase-discordant SNPs were annotated in relation to disease by use of GWA Studies, the GAD and OMIM data obtained from UCSC hg18 table browser.

### Analysis of unique gene haplotypes and diplotypes

To determine the number of unique molecular haplotypes and diplotypes, we utilized the ReFHap output data generated for each individual genome, containing all phased SNP alleles linearly ordered by their genomic coordinates (hg18). First, these ReFHap output data were merged into an integrated data set of 14 molecularly haplotype-resolved genomes. This data set was then consolidated into a haplotype input matrix, with each row representing a heterozygous coordinate and each column representing one of two phased nucleotides of an individual genome. Thus, two adjacent columns contain the unique combinations of nucleotides that characterize the haplotype sequences of a given genome. The haplotype input files downloaded from the 1000G database were structured identically. Then, the heterozygous coordinates were intersected with the RefSeq (hg18) gene coordinates to select the heterozygous positions within primary transcripts. Corresponding 1000G files were filtered accordingly. This allowed, for each gene, immediate extraction of a sub-matrix containing all phased haplotypes of this gene in rows, providing the basis to extract its different haplotypes. Only those genes were included in cross-subject comparisons that had full haplotype counts in 5, 10 and 14 molecularly resolved genomes. A haplotype was defined as being ‘different’, or unique, if its nucleotide sequence differed in at least one position from the nucleotide sequence of all other haplotypes. We sorted the rows by alphabetic identity and removed the duplicates to obtain a list of the unique haplotypes for each gene.

The unique pairs of haplotypes (diplotypes, definition 1) were determined analogously. To this end, the two haplotypes of each gene were combined to form one linear nucleotide sequence. This allowed generation of a gene level diplotype matrix, with each row representing one diplotype. A diplotype was defined as unique, if its combined nucleotide sequence differed in at least one position from all other diplotypes. Then each diplotype was compared with all of the other diplotypes of the gene and duplicates were marked. Since the two haplotypes of a diplotype can be combined into one linear sequence in two ways, both possible combinations were evaluated and subjected to comparisons. Duplicate entries were removed to extract the list of unique diplotypes for each gene in defined number of genomes.

To estimate the potential impact of switch errors in molecular and statistical phasing on the quantification of unique haplotypes and diplotypes, we have (i) assessed the distribution of existing phase-discordant sites across all genes in the molecular data set, and (ii) assessed the probability that genes are assigned false statistical haplotypes, based on a median ‘switch distance’ of 300 kb described for 1000G haplotypes^11^ (details in Supplementary Methods).

To estimate the potential impact of missing data in the case of incompletely molecularly haplotype-resolved genomes, we have tested and corroborated the quantification of haplotype diversity in simulation studies. These showed that fractions of 75, 50, 25 and 5% of all genes still produce representative results, expressed as percentages relative to total input count (deviation in the case of missing data at most by 0.7%) or as averages across given gene counts (deviation at most by 0.1%). As an inherent feature of fosmid-based methodology, the genomic regions that are phased may differ between genomes and the number of genes for which phase is simultaneously available across genomes decreases for increasing number of genomes^41^.

Once the unique haplotypes and diplotypes were determined for each gene, we assessed their FoO. These were derived from the gene level haplotype/diplotype matrices, counting the duplicate rows and calculating their fractions of total haplotype/diplotype input rows. The assessment of FoOs provided in a first pass the basis to classify autosomal genes into three distinct categories based on their frequency spectra. Then, the FoOs of the major, common and un-common haplotypes were calculated separately for each of these three categories.

### Analysis of unique protein haplotypes and diplotypes

Analyses were performed analogous to those described for the gene haplotypes, using the subset of nsSNPs that cause AA exchanges; thus, ‘protein’ haplotypes and diplotypes refer to protein-coding sequences and pairs thereof. Averages ‘per gene’ were calculated per total number of protein-coding sequences assessed, and per variable protein sequences (shown in Supplementary Table 12c).

### Population fit and extrapolation

To extrapolate the number of unique genes and protein haplotypes and diplotypes (definition 1) to much larger population sizes, we approximated the data values for 10,000 up to 1 million genomes with a power function, based on the available data for 5 up to 372 genomes. After fitting the datapoints for 5, 10, 14, 20, 40, 57, 200, 372 and 628 genomes, the number of unique gene haplotypes *y*_a_ were approximated with the function *y*_a_=97.006 × ^−0.1803^, *R*^2^=0.96 and the number of unique gene diplotypes *y*_b_ with the function *y*_b_=104.87 × ^−0.0836^, *R*^2^=0.99. The number of unique protein haplotypes *y*_c_ were approximated with the function *y*_c_=38.346 × ^−0.640^, *R*^2^=0.95 and the number of unique protein diplotypes *y*_d_ with the function *y*_d_=70.173 × ^−0.547^, *R*^2^=0.96. The numbers for one million genomes were evaluated in addition under assumption of phasing (switch) errors (Supplementary Methods).

### Extraction of CDP

All key steps to derive the CDP have been outlined in main text. First, we scored all genes in each genome as ‘protein diplotype’ that had at least one nsSNP. The resulting genome level data sets were merged into a sample level set of genes encoding protein diplotypes, and the diplotype frequency (diplotype counts per total genome count) for each gene derived. Subsequent steps are as described. To intersect 57CEU and 372EUR, we routinely subtracted 57CEU from 372EUR as a subset thereof to take advantage of the largest available European sample. The genes that were shared by either intersection (14G with 57CEU and 57CEU with 372EUR) at the threshold with the strongest overlap (30%) were integrated into one gene set, the CDP. Thus, this set contained genes that were validated by presence in at least two of the three data sets analyzed. The sequential procedure of analyzing overlaps appeared preferable to avoid 14G being the limiting factor for the extraction of a CDP, while on the other side utilizing the advantage of both molecular and statistical data sources. For additional validation, we intersected the CDP with the subset of genes that encoded protein diplotypes in at least 30% of 372EUR, resulting in an overlap of 91%. Simulation studies were performed to validate the CDP against chance occurrence (Supplementary Methods) and derive statistical significance values validating the cutoff at a diplotype frequency threshold of 30%.

### Analysis of phase configurations

To analyze the phase configurations of potentially damaging mutations and their distribution at the whole-genome level, we first applied PolyPhen-2 (ref. 15) and SIFT^14^ to the ReFHap output files generated from each of the 14 molecularly phased genomes. The resulting intermediate output files had the mutations denoted by 1 (alleles different from the reference sequence)^4^,^5^, and the reference sequence alleles denoted by 0. The specific combinations of mutations were contained in two adjacent columns representing ‘Haplotype 1’ and ‘Haplotype 2’, with the rows representing the heterozygous coordinates and their gene IDs. Corresponding 1000G-derived files for 57CEU and 372EUR were filtered for potentially damaging mutations using the 1000G annotation information described above and intermediate output files prepared accordingly.

To assess the concrete phase configurations, we used and automated the following approach: column 1 representing ‘Haplotype 1’ was examined, moving 5′ to 3′ from cell to cell, each containing allele 1 or 0 assigned to a genomic coordinate and gene ID. Where only one cell was assigned to a certain gene ID, a gene had only one potentially damaging mutation and therefore was removed, ensuring that only those mutations that required phasing were evaluated Then, the series of alleles across all cells assigned to the same gene ID were stored as units and subjected to assessment of phase configuration. If all stored alleles in column 1 were solely 1s or 0s, a *cis* configuration was scored; otherwise a *trans* configuration was scored. This procedure generated a result file for each genome, which contained the gene IDs with *cis* or *trans* assigned, allowing immediate calculation of the *cis*/*trans* ratio and the average ratios across 14G, 57CEU and 372EUR.

To examine the distribution of *cis* and *trans* configurations at the single gene level, the genome level result files were merged into sample level data sets, and the number of *cis* and *trans* configurations were assessed separately for each of the 17,861 RefSeq genes. To evaluate the relative abundance of either configuration, the difference between *cis* and *trans* counts was chosen as a measure, because it appeared more robust than the ratio of *cis* to *trans*. The latter proved more vulnerable to small deviations in the divisor. Moreover, a binomial approach was tested, calculating for each gene the probability (binominal distribution) of deviating from a random distribution of *cis* and *trans* configurations, leaving most of the genes (73.5%) abundant for either configuration. *Cis*- or *trans*-abundant genes were distinguished based on difference values as described in main text. To create an as robust as possible basis for further evaluation of *cis*- versus *trans*-abundant genes, the three sample level data sets were combined to a superset, compiling a list of 4,757 genes with their *cis* and *trans* counts assigned. This list served as input for the Wilcoxon rank test^23^ to perform GO enrichment analysis with a preference for either end of the spectrum without setting a concrete cutoff criterion. Then the 4,757 genes were sorted by difference (*cis–trans*) and 200 genes (manually chosen threshold of ~5% from 4,757 genes) extracted from both ends. These were used as input for pathway analysis.

### Analysis of phase differences

Phase differences between genomes and genes were assessed at shared heterozygous sites (Supplementary Note 7). To begin with, we performed pair-wise genome comparisons in 372EUR to identify pairs of identical potentially damaging mutations that resided in both *cis* and *trans* configurations. To this end, all phased heterozygous SNPs in each genome were filtered for mutations using the annotation information at ftp.1000genomes.ebi.ac.uk/vol1/ftp/release/20100804/. Then we performed all possible pair-wise genome comparisons (0.5 × 372^2^=69,192 comparisons) to determine the pair-wise overlaps of mutations by intersecting their chromosomal coordinates. Thus, the resulting 69,192 intermediate data sets contained, for each pair of genomes, the chromosomal coordinates of their shared mutations in row with their phase information, with 1s denoting the perturbing mutations (different from the reference sequence) and 0s the reference alleles. Columns 1 and 2 contained ‘Haplotype 1’ and ‘Haplotype 2’ of the first, and columns 3 and 4 contained both haplotypes of the second of the intersected genomes. Then, to assess phase differences within each of these intermediate data sets, a sliding window approach was applied (5′− 3′) with a window size of two mutations. Within the window, phase information from the first genome was compared with phase information from the second, evaluating columns 1 and 3: Where one column contained only 1s or 0s, and the other both 1 and 0, the pair of mutations was recorded as phase different, with their chromosomal coordinates assigned (median distance between two potentially damaging mutations 3.6 Mb). In the next step, all these pairs were merged into a sample level data set where the entirety of phase-different mutation pairs was aligned against their genomic coordinates 5′–3′. This data set was filtered to contain only mutation pairs that were observed twice. Finally, the entirety of phase-different pairs of mutations was condensed (removing all duplicate entries) into a sorted list of anchor mutations (5′ mutations), each with their adjacent 3′ ‘phase-alternate’ mutations arrayed in one row. The set of ‘phase-alternate’ genes was extracted by selecting all phase-different mutation pairs that had both their chromosomal coordinates within the exon boundaries of the same gene. Notably, in roughly 28% of these genes, one or more additional perturbing, nonshared mutations were present.

### Biological analysis methods (II)

GO-Annotation for all genes was downloaded from Biomart (February 2012). For gene sets of interest, a GO group enrichment analysis was performed using FUNC (https://func.eva.mpg.de/)^23^, choosing the hyper-geometric test, a cutoff of at least five genes per group, and 1,000 permutations. If the global test indicated significant enrichment, the refinement was executed for GO groups that were significant before refinement with *P*<0.05, and reporting GO groups that were significant after refinement with *P*<0.05. To test for enrichment of disease genes we utilized the Gene Set Enrichment Analysis (http://www.broadinstitute.org/gsea/index.jsp)^38^. We only chose the category ‘curated gene sets’, which contains sets of genes that have been associated with certain diseases. We report disease gene sets that are enriched in our gene lists of interest with *P*<0.05. Input data for enrichment analyses of categories 1, 2 and 3 genes were not normalized for gene length^39^ (Supplementary Note 3).

For exploring functional content of gene sets of interest, an over-representation analysis was performed with human molecular pathways using the ConsensusPathDB tool (CPDB, http://consensuspathdb.org, release 26 (ref. 40). Gene sets of 200 *cis*-abundant and 200 *trans*-abundant genes, serving as input, were analyzed setting a threshold of *P*<0.001 (hyper-geometric test). To filter out unspecific enrichment, we only took pathways into account with minimum overlap of five genes with the gene sets. As the background set, we used 17,861 RefSeq genes^36^ downloaded from the UCSC table browser (hg18). Only chromosomes 1–22 were included in the analyses. The input gene set representing the CDP was analyzed requiring a minimum overlap of 20 genes. This accounts for the large number of input genes (4,269) in this gene set to avoid unspecific enrichment with larger pathways. Corrections for multiple testing were integrated in all described programmes applied to GO and pathway analysis.

### Data access

Sequence alignments and haplotypes for all molecularly phased genomes are available from the website http://www.molgen.mpg.de/~genetic-variation/ngs_data/. In addition, haplotypes for all molecularly phased genomes can be viewed in a UCSC browser session at http://www.molgen.mpg.de/~genetic-variation/UCSC_12phasedgenomes. Data files related to gene categories, the ‘CDP’ and ‘phase-alternate’ genes can be downloaded from http://www.molgen.mpg.de/~genetic-variation/diploid_landscape/.

## Author contributions

M.R.H. conceived and conducted the study, wrote the manuscript, guided and supervised the bioinformatic analyses, and jointly with E.-K.S. designed and supervised the molecular genetics work. G.M.C. and H.L. provided critical input and discussion of content and analyses. T.K. established key steps for fosmid library production. E.-K.S., S.P. and S.S. established next generation sequencing platform and experimental methods and carried out wet lab production. K.N. conducted enrichment analyses. T.H. designed and implemented main parts of the bioinformatics pipeline and performed the bulk analysis of data. All authors provided comments and input to the manuscript.

## Additional information

**How to cite this article:** Hoehe, M. R. *et al*. Multiple haplotype-resolved genomes reveal population patterns of gene and protein diplotypes. *Nat. Commun.* 5:5569 doi: 10.1038/ncomms6569 (2014).

**Accession Codes**. Short read sequence data from the twelve molecular haplotype-resolved genomes have been deposited in the European Nucleotide Archive (ENA) hosted by EMBL, under the accession code PRJEB7549. Sequence data from MP1 and NA12878 have been deposited in the ENA under the accession codes ERP000494 and ERP000819, respectively.

## Supplementary Material

Supplementary Information Supplementary Figures 1-4, Supplementary Tables 1-17, Supplementary Notes 1-7, Supplementary Methods and Supplementary References

## Figures and Tables

**Figure 1:**
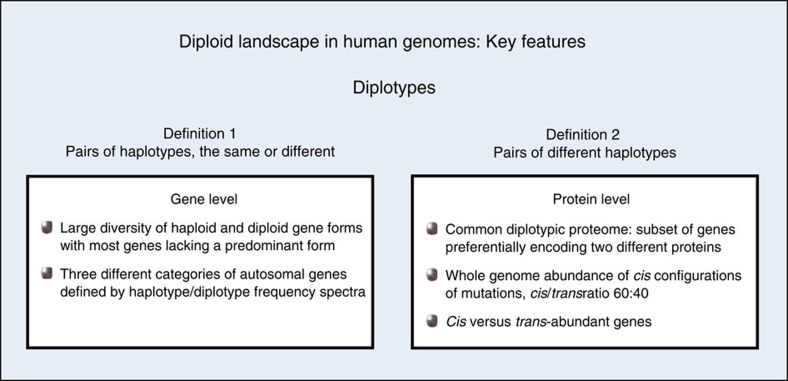
Overview of key features characterizing the diploid landscape. A summary of the key features describing the diplotypic nature of human genomes in European population samples is presented in context with the specific definitions of ‘diplotypes’. Gene and protein level analyses are distinguished.

**Table 1:** Whole-genome *cis*-abundance of mutations.

**Set of phased genomes**	***Cis*** configurations (%)*	***Trans*** configurations (%)*	**s.d.**	***P*****-value**†
*Potentially perturbing mutations*‡§				
14G||	64.1	35.9	5.7	2.11 × 10^−3^
57CEU¶	60.4	39.6	2.9	7.45 × 10^−5^
372EUR¶	61.7	38.3	2.1	7.68 × 10^−8^
*Non-synonymous SNPs*§				
14G||	59.6	40.4	2.9	1.33 × 10^−5^
57CEU¶	58.1	41.9	1.8	5.98 × 10^−7^
372EUR¶	57.0	43.0	1.3	4.67 × 10^−7^

SNP, single nucleotide polymorphism.

^*^Mean value.

^†^Exact binomial test.

^‡^Predicted by use of PolyPhen-2 (ref. 15) and SIFT^14^.

^§^Autosomal RefSeq hg18 genes from UCSC table browser.

^||^Haplotype-resolved by application of fosmid pool-based next generation sequencing^4^.

^¶^Statistically haplotype-resolved genomes from 1000 Genomes Project^11^,^16^.
