# Supplementary material for: Multiple haplotype-resolved genomes reveal population patterns of gene and protein diplotypes
Source: Nat Commun. 2014 Nov 26;5:5569. doi: 10.1038/ncomms6569 (PMC4263165; doi:10.1038/ncomms6569)
Supplement: Supplementary Information — Supplementary Figures 1-4, Supplementary Tables 1-17, Supplementary Notes 1-7, Supplementary Methods and Supplementary References [file ncomms6569-s1.pdf]

Multiple haplotype-resolved genomes reveal population patterns of gene and protein diplotypes

Supplementary Figures

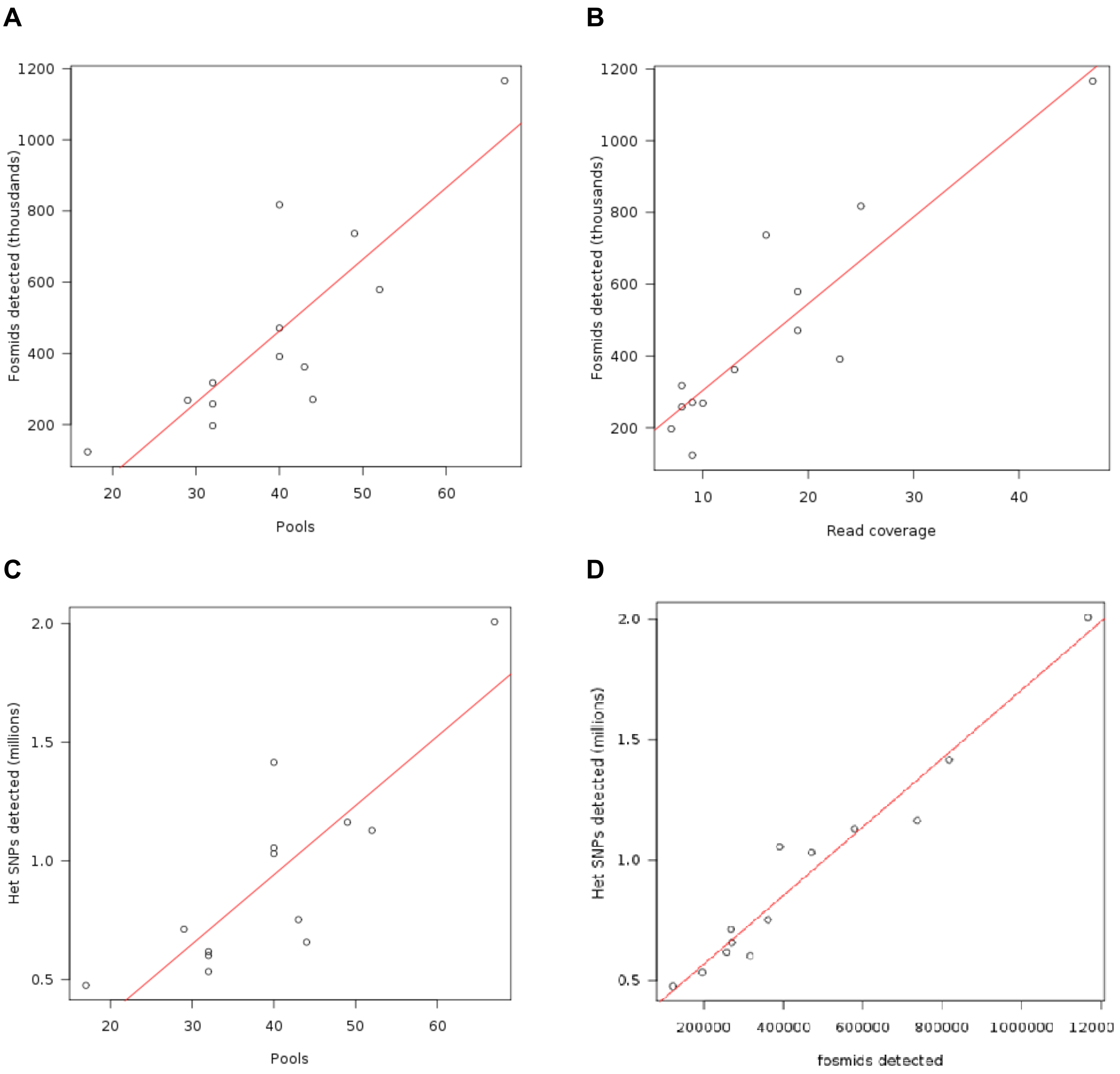

Supplementary Figure 1 Detection of fosmids and heterozygous SNPs as a function of NGS production

The numbers of fosmids detected are presented (A) in relation to the numbers of fosmid pools sequenced, and (B) the read coverage obtained. The numbers of heterozygous SNPs detected are presented (C) in relation to the numbers of fosmid pools sequenced, and (D) the numbers of fosmids detected. Data points for MP1[4] are included.

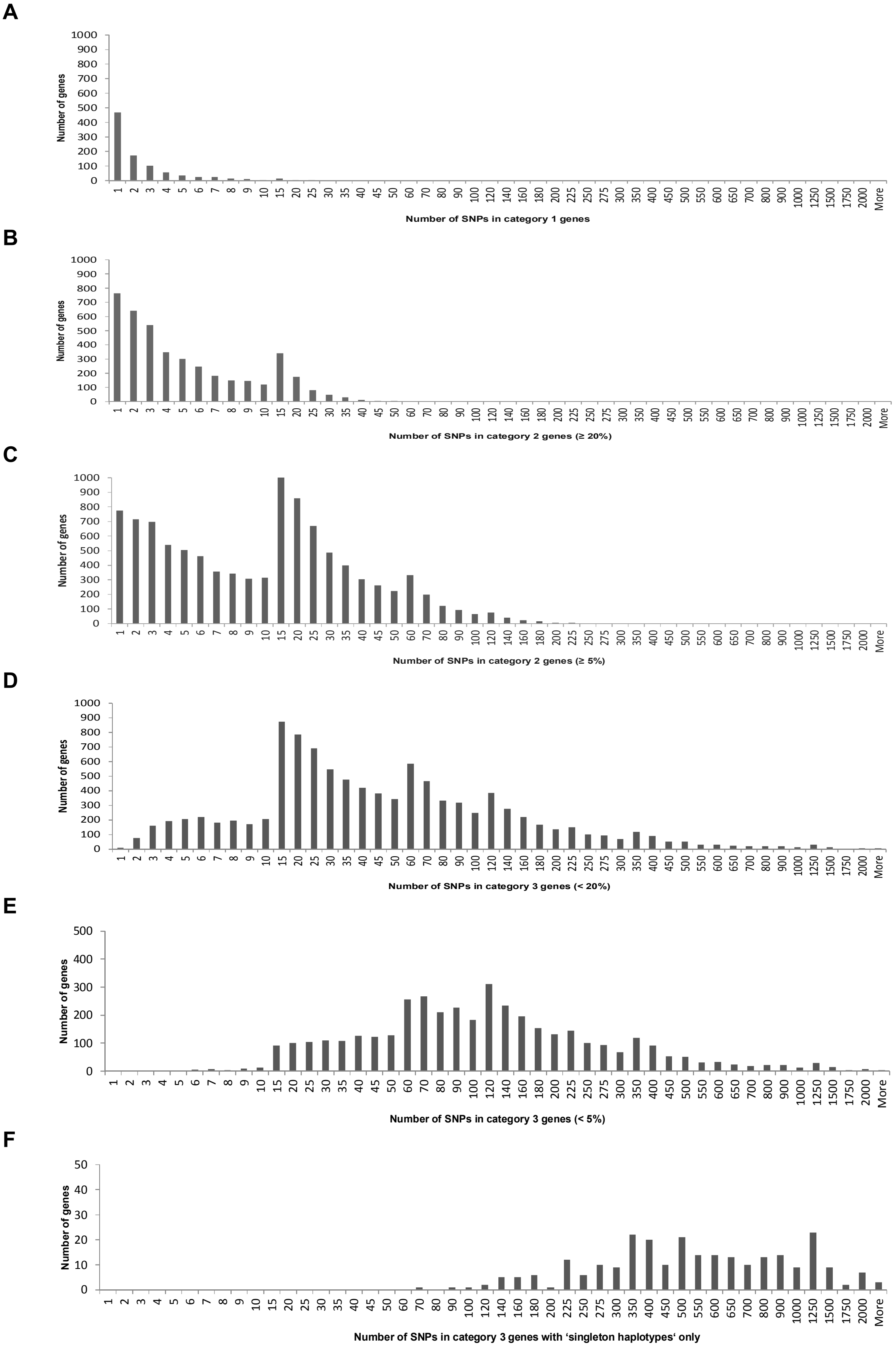
A
Number of genes
Number of SNPs in category 1 genes
B
Number of genes
Number of SNPs in category 2 genes (≥ 20%)
C
Number of genes
Number of SNPs in category 2 genes (≥ 5%)
D
Number of genes
Number of SNPs in category 3 genes (< 20%)
E
Number of genes
Number of SNPs in category 3 genes (< 5%)
F
Number of genes
Number of SNPs in category 3 genes with 'singleton haplotypes' only

G

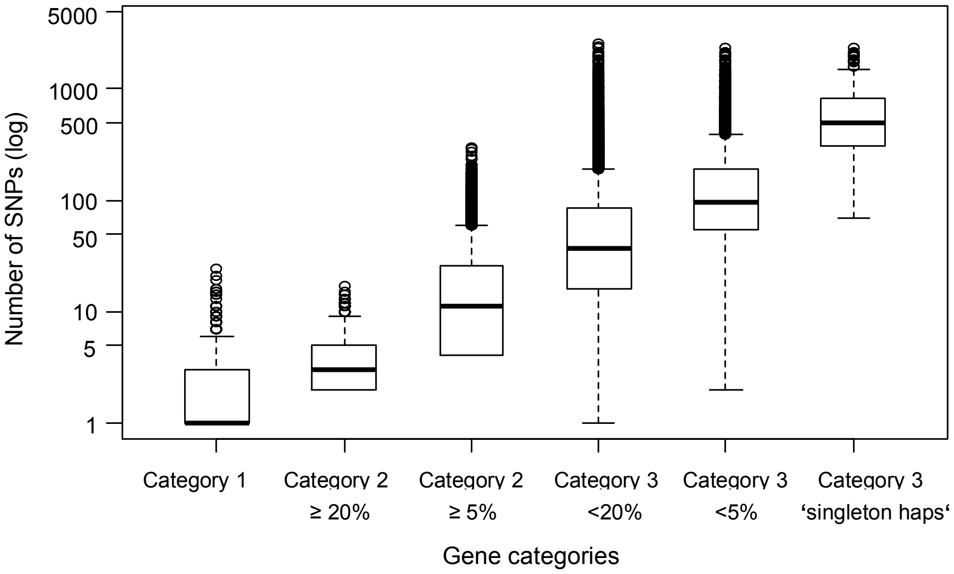

H

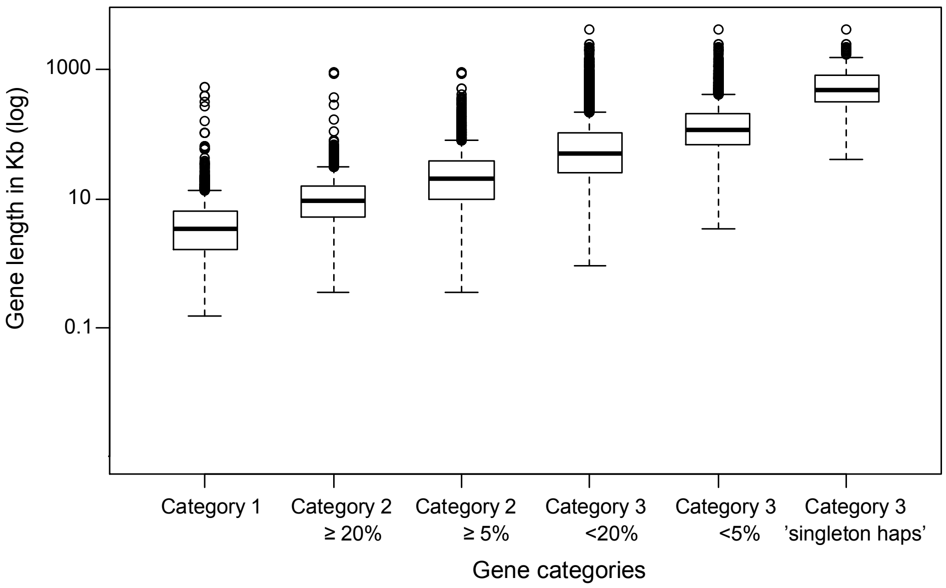

Supplementary Figure 2 Distinction of category 1, 2 and 3 genes by SNP profiles

In these histograms, each dark grey bar indicates the numbers of genes containing specified numbers of SNPs in the sample set of 57CEU. When exceeding 10, SNP numbers are binned into increasingly larger intervals. Thus, jumps in the curves are due to binning. Gene categories are defined as follows: Category 1 genes by presence of one major/predominant haplotype with a frequency of occurrence (FoO) ≥50%; category 2 genes by at least one common haplotype with a FoO ≥20% (results for ≥5% shown in addition). Category 3 genes have only haplotypes below a FoO of 20%; results for ≤5% and the subset of category 3 genes consisting of 'singleton haplotypes' only are shown in addition. **(A)** Histogram of category 1 genes; **(B)** category 2 genes (frequency threshold ≥20%); **(C)** category 2 genes (frequency threshold ≥5%); **(D)** category 3 genes (frequency threshold <20%); **(E)** category 3 genes (frequency threshold <5%); **(F)** subset of category 3 genes encoding 'singleton haplotypes' only. **(G)** As a summary, box plots for these gene categories are shown for n=57. The horizontal line inside each box marks the median. The lower and upper hinges correspond to the 25th and 75th percentiles. The lower and upper whiskers extend to the lowest and highest values that are within 1.5 times inter-quartile range; outliers beyond this range are plotted as points. , indicating median, upper and lower quartiles, minimum, maximum and outlier SNP numbers, are shown. **(H)** Summary box plots represented accordingly to (G) for gene length (kb).

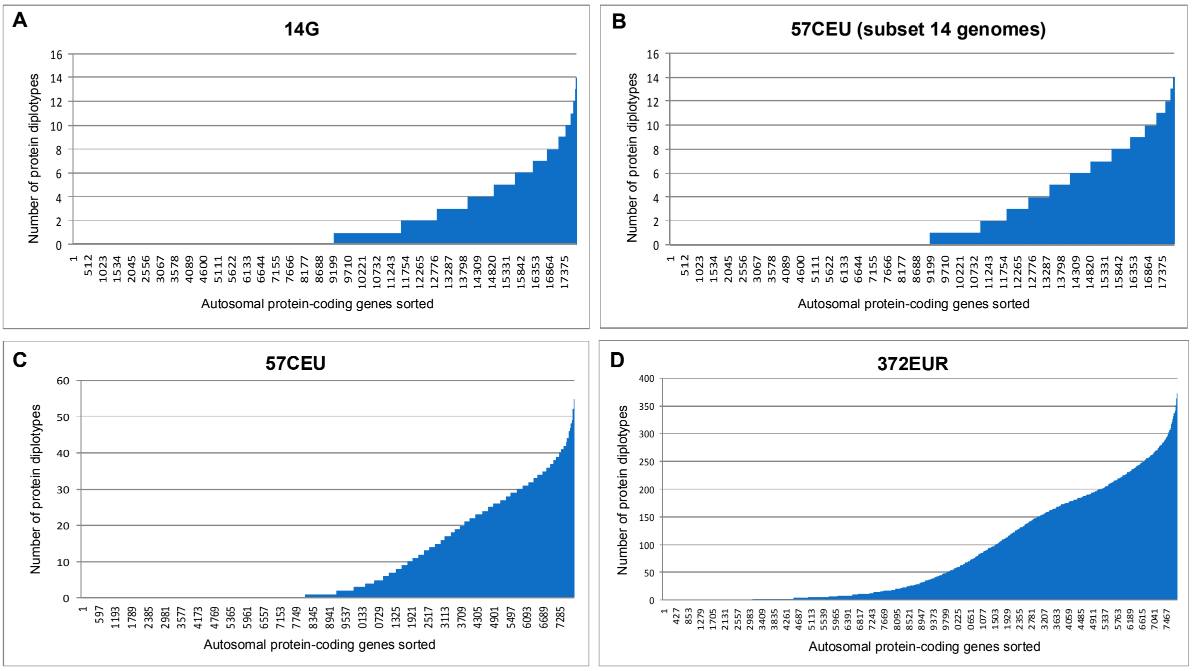

Supplementary Figure 3 Subsets of autosomal genes encoding protein diplotypes

Genes sorted by increasing numbers of protein diplotypes counted in the set of 14 molecularly haplotype-resolved genomes (14G) **(A)**, a corresponding subset extracted from 57CEU **(B)**, the total set of 57CEU **(C)** and 372EUR statistically resolved genomes **(D)** from 1000 Genomes Project database[16,11]. The *x* axis indicates the numbers of (sorted) autosomal protein-coding genes, the *y* axis the numbers of protein diplotypes. Protein diplotype defined by presence of at least one nsSNP.

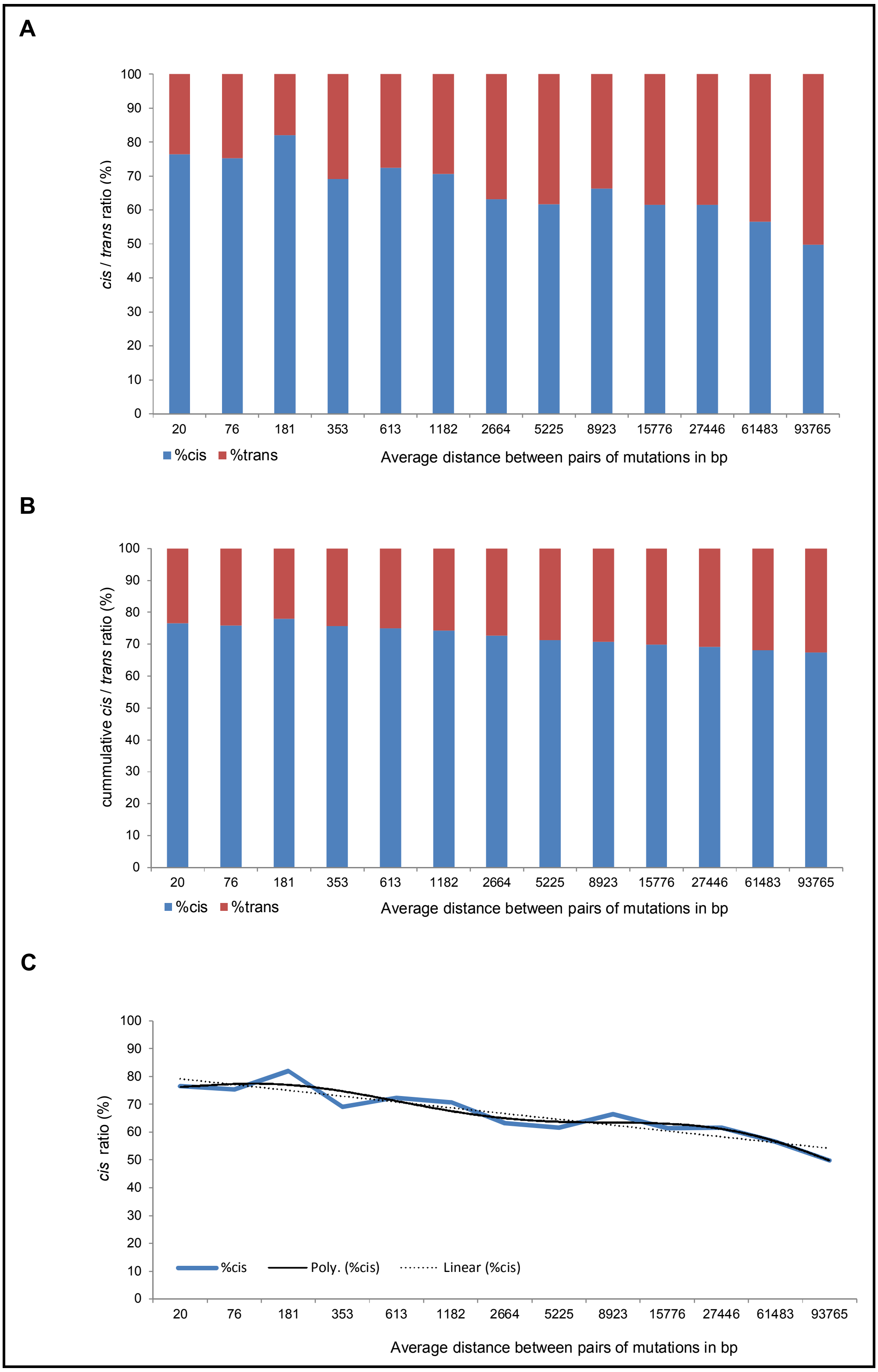

Supplementary Figure 4 Relationship between inter-mutation genomic distance and *cis*/*trans* ratios

The percentages (%) of *cis* configurations (blue bars) and *trans* configurations (red bars) are presented in relation to average values of inter-mutation distances. Each bin represents 10,000 *cis* or *trans* configurations (*cis* and *trans* configurations were sorted by inter-mutation distances and binned per 10,000 configurations). **(A)** Fractions of *cis* and *trans* configurations per bin; **(B)** Cumulative fractions of *cis* and *trans* configurations across bins of increasing inter-mutation distance; **(C)** Regression analysis based on polynomial and linear approximation for the *cis* ratio.

Supplementary Tables

Supplementary Table 1 Data summary for the sequenced fosmid pools[1]

Subject	No. fosmid pools	Uniquely mapped bases (Gb)	Uniquely mapped reads	Mean read coverage	Mean haploid read coverage	No. fosmids detected
**MP2**	40	63	1,259,631,402	25	12.5	817,503
**MP3**	49	47	938,609,268	16	8	736,910
**MP4**	52	50	1,001,119,890	19	9.5	579,305
**MP5**	40	54	1,075,854,620	23	11.5	391,133
**MP6**	40	48	952,166,323	19	9.5	471,030
**MP7**	43	31	619,330,764	13	6.5	361,925
**MP8**	32[2]	29	570,341,505	10	5	268,273
**MP9**	44	23	459,427,159	9	4.5	270,558
**MP10**	32	24	484,049,568	8	4	257,968
**MP11**	32	21	421,191,889	8	4	317,434
**MP12**	32	20	395,886,226	7	3.5	196,798
**MP13**	32[2]	21	426,994,961	10	5	123,261
**Avg.**	39	36	717,050,298	14	7	399,342

[1] Fosmid pools contained ~15,000 fosmids, representing ~15% of the diploid genome (Supplementary Methods).
[2] Few low-complexity fosmid pools were excluded from analysis.

Supplementary Table 2 SNP calling accuracy[1]

Subject	No. false negatives[2]	%[3]	No. false positives[4]	%[3]	Total No. false negatives & false positives[5]
**MP2**	2,455	1.05	847	0.50	3,302
**MP3[6]**	-	-	-	-	
**MP4**	7,263	3.15	431	0.25	7,694
**MP5**	12,686	5.58	628	0.36	13,314
**MP6**	15,549	6.50	560	0.34	16,109
**MP7**	7,440	3.16	332	0.19	7,772
**MP8**	22,368	9.74	373	0.21	22,741
**MP9**	6,920	2.90	269	0.15	7,189
**MP10**	11,005	4.79	178	0.10	11,183
**MP11**	6,200	2.67	244	0.14	6,444
**MP12**	14,314	5.95	201	0.12	14,515
**MP13**	22,199	9.58	142	0.08	22,341
**Avg.**	12,594	5.40	336	0.19	12,930

[1] Fosmid-based SNP calling by use of SNVQ (Supplementary Methods) was compared to Affy 1000K genotypes.
[2] Sum of all calls that were discordant between fosmid-based SNP calling and Affy 1000K genotypes: where SNVQ does not call a heterozygous or homozygous (different from the reference) SNP in the presence of a heterozygous or homozygous Affy 1000K genotype[1]. False negatives do not include SNPs in unphased DNA (19% up to 60% of the genomes).
[3] Per total calls.
[4] Sum of discordant calls where SNVQ calls either a heterozygous or homozygous (different from the reference) SNP.
[5] Total number of het/hom discrepancies between the SNVQ and Affy 1000K genotype data.
[6] No Affy 1000K genotype data available.

Supplementary Table 3 Molecular phasing results from 12 European genomes

A. Phasing and SNP data

Subject	Molecularly phased contigs					SNPs detected and phased[1]				
	No. contigs	Bases in phased contigs	% total phased	N50[2] length (bp)	Max contig length (bp)	All SNPs	Het SNPs	Het SNPs phased	% het SNPs phased	SNPs in phase context[3]
**MP2**	8,064	2,179,089,606	81.3	628,898	5,163,406	2,440,123	1,414,752	1,388,000	98.1	2,098,556
**MP3**	9,101	2,016,157,683	75.2	476,471	2,451,048	1,991,571	1,162,750	1,148,010	98.7	1,733,977
**MP4**	10,113	1,964,364,681	73.3	401,459	2,824,287	2,147,156	1,128,402	1,113,917	98.7	1,722,378
**MP5**	14,172	1,822,059,454	68.0	252,221	2,411,857	2,142,682	1,054,723	1,033,337	98.0	1,655,127
**MP6**	13,369	1,848,496,240	69.0	267,416	2,063,559	2,070,810	1,030,636	1,009,625	98.0	1,618,651
**MP7**	16,518	1,564,345,995	58.3	184,509	1,579,792	1,536,072	752,602	701,769	93.2	1,078,494
**MP8**	16,474	1,544,331,130	57.6	168,529	3,147,226	1,719,358	712,584	688,560	96.6	1,192,964
**MP9**	18,593	1,477,595,969	55.1	145,698	1,314,557	1,295,951	657,973	592,085	90.0	926,399
**MP10**	17,669	1,471,968,874	54.9	148,798	947,977	1,407,538	618,033	579,261	93.8	938,839
**MP11**	15,962	1,520,917,679	56.7	178,814	1,993,929	1,282,743	601,596	547,718	91.0	857,370
**MP12**	21,407	1,303,431,537	48.6	102,104	818,941	1,274,561	533,961	475,854	89.1	794,563
**MP13**	23,386	1,062,937,598	39.6	73,069	721,226	1,206,705	476,169	411,173	86.3	713,228

[1] Reference is NCBI Build 36.1 un-gapped lengths. Only autosomes phased.
[2] N50 value: 50% of the covered bases are found within contigs longer than the given number.
[3] Total number of heterozygous and homozygous non-reference SNPs within phased contigs.

B. Summary of autosomal genes phased

Subject	No. phased genes[1]	%[1]	No. het SNPs in phased genes
**MP2**	12,976	72.7	335,803
**MP3**	11,292	63.2	238,735
**MP4**	11,005	61.6	236,411
**MP5**	10,376	58.1	191,321
**MP6**	10,361	57.9	195,680
**MP7**	8,938	50.0	128,024
**MP8**	7,115	39.8	84,673
**MP9**	7,262	40.6	81,997
**MP10**	6,408	35.8	63,879
**MP11**	7,485	41.8	78,717
**MP12**	5,430	30.4	45,832
**MP13**	4,480	25.1	34,756

[1] Relative to total number of autosomal RefSeq (hg18) genes from UCSC table browser.

Supplementary Table 4 Characterization of heterozygous SNPs

Subject	No. het SNPs	No. novel SNPs[3]	%[1]	No. het SNPs in genes	%[1]	No. het SNPs 10kb upstream	%[1]	No. het SNPs exons	%[1]	No. nsSNPs	%[1]	No. damaging mutations[2]	%[1]	GWA SNPs	%[1]
**MP2**	1,414,752	30,937	2.19	510,674	36.1	79,572	5.6	20,803	1.5	5,352	0.38	1,630	0.12	1,478	0.10
**MP3**	1,162,750	17,668	1.52	413,395	35.6	61,554	5.3	16,619	1.4	4,225	0.36	1,359	0.12	1,117	0.10
**MP4**	1,128,402	17,755	1.57	419,101	37.1	62,769	5.6	17,345	1.5	4,285	0.38	1,345	0.12	1,306	0.12
**MP5**	1,054,723	28,540	2.71	387,164	36.7	62,405	5.9	16,706	1.6	4,325	0.41	1,358	0.13	1,212	0.11
**MP6**	1,030,636	14,536	1.41	389,198	37.8	60,122	5.8	16,413	1.6	4,133	0.40	1,283	0.12	1,262	0.12
**MP7**	752,602	6,208	0.82	295,153	39.2	46,795	6.2	13,339	1.8	3,299	0.44	1,049	0.14	1,100	0.15
**MP8**	712,584	7,691	1.08	258,203	36.2	37,348	5.2	10,595	1.5	2,716	0.38	817	0.11	1,072	0.15
**MP9**	657,973	2,896	0.44	254,494	38.7	37,651	5.7	10,758	1.6	2,593	0.39	765	0.12	1,106	0.17
**MP10**	618,033	2,783	0.45	227,986	36.9	31,910	5.2	9,399	1.5	2,304	0.37	696	0.11	931	0.15
**MP11**	601,596	2,014	0.33	229,009	38.1	33,690	5.6	9,970	1.7	2,510	0.42	746	0.12	965	0.16
**MP12**	533,961	2,873	0.54	201,430	37.7	29,217	5.5	8,209	1.5	2,020	0.38	607	0.11	889	0.17
**MP13**	476,169	3,018	0.63	185,003	38.9	27,404	5.8	8,217	1.7	2,013	0.42	598	0.13	886	0.19

[1] Relative to total number of heterozygous SNPs.
[2] Predicted by PolyPhen-2[2] and SIFT[3].
[3] Number of novel SNPs per individual also depend on number of detected fosmids and mean read coverage (variation presumably due to differences in the numbers of detected fosmids, mean read coverage, complexity of sequenced fosmid pools and individual biological differences).

Supplementary Table 5 Overview of molecular diplotypes in autosomal protein-coding genes in 12 European genomes

A. Gene level

	Transcripts						Transcripts + 10kb upstream						10kb upstream					
	≥1 het SNP[1]		≥2 het SNPs[2]		phased		≥1 het SNP		≥2 het SNPs		phased		≥1 het SNP		≥2 het SNPs		phased	
Subject	No.	%	No.	%	No.	%[3]	No.	%	No.	%	No.	%[3]	No.	%	No.	%	No.	%[3]
MP2	14,510	84.1	12,645	73.3	9,852	77.9	16,321	94.6	14,877	86.2	11,474	77.1	13,397	77.7	10,826	62.8	10,306	95.2
MP3	13,693	78.7	11,675	67.1	8,337	71.4	15,611	89.7	13,953	80.2	9,809	70.3	12,422	71.4	9,600	55.2	9,005	93.8
MP4	13,713	78.2	11,748	67.0	8,263	70.3	15,533	88.5	13,893	79.2	9,607	69.1	12,184	69.4	9,523	54.3	8,879	93.2
MP5	13,891	83.4	11,817	70.9	7,605	64.4	15,780	94.7	14,098	84.6	8,935	63.4	12,271	73.7	9,535	57.2	8,694	91.2
MP6	13,724	79.3	11,638	67.2	7,517	64.6	15,649	90.4	13,922	80.4	8,819	63.3	12,121	70.0	9,266	53.5	8,427	90.9
MP7	13,103	83.4	10,973	69.9	6,350	57.9	14,999	95.5	13,240	84.3	7,504	56.7	11,404	72.6	8,533	54.3	7,415	86.9
MP8	12,056	79.5	9,842	64.9	4,708	47.8	14,108	93.0	12,065	79.5	5,658	46.9	9,939	65.5	6,861	45.2	5,699	83.1
MP9	12,410	77.1	10,223	63.5	4,941	48.3	14,414	89.6	12,490	77.6	5,860	46.9	10,658	66.2	7,630	47.4	6,069	79.5
MP10	11,916	80.5	9,680	65.4	4,242	43.8	13,907	94.0	11,886	80.3	5,099	42.9	9,760	66.0	6,713	45.4	5,316	79.2
MP11	12,268	69.5	10,168	57.6	5,111	50.3	14,214	80.6	12,276	69.6	6,109	49.8	10,266	58.2	7,233	41.0	5,941	82.1
MP12	11,510	80.9	9,311	65.4	3,414	36.7	13,542	95.2	11,478	80.7	4,079	35.5	9,390	66.0	6,309	44.3	4,530	71.8
MP13	11,210	83.0	9,014	66.8	2,727	30.3	13,241	98.1	11,070	82.0	3,195	28.9	8,806	65.2	5,630	41.7	3,727	66.2

[1] All genes that contain at least one het SNP and so have two different molecular forms.
[2] All genes that contain at least two het SNPs and therefore require molecular phasing.
[3] Relative to total number of transcripts/regions containing ≥2 het SNPs; phased genes/regions are entirely contained within phased sequence.

B. Protein level

Subject	Genes assessed[1]	Protein-coding sequences											
		≥1 AA exchange		≥2 AA exchanges		phased		≥1 damaging AA exchange[2]		≥2 damaging AA exchanges[2]		phased	
	No.	No.	%	No.	%	No.	%[3]	No.	%	No.	%	No.	%[3]
**MP2**	15792	3,190	20.2	903	5.7	775	85.8	1,278	8.1	208	1.3	175	84.1
**MP3**	14758	2,627	17.8	680	4.6	545	80.1	1,095	7.4	159	1.1	125	78.6
**MP4**	16453	2,649	16.1	700	4.3	553	79.0	1,033	6.3	156	0.9	127	81.4
**MP5**	11677	2,604	22.3	690	5.9	512	74.2	1,056	9.1	167	1.4	125	74.9
**MP6**	15665	2,522	16.1	678	4.3	535	78.9	994	6.4	147	0.9	116	78.9
**MP7**	12861	2,225	17.3	521	4.1	361	69.3	869	6.8	118	0.9	81	68.6
**MP8**	11100	1,776	16.0	402	3.6	245	60.9	672	6.1	83	0.7	52	62.7
**MP9**	10478	1,865	17.8	355	3.4	211	59.4	659	6.3	73	0.7	41	56.2
**MP10**	9005	1,639	18.2	309	3.4	186	60.2	603	6.7	56	0.6	31	55.4
**MP11**	9471	1,809	19.1	356	3.8	227	63.8	648	6.8	72	0.8	43	59.7
**MP12**	6587	1,469	22.3	286	4.3	132	46.2	545	8.3	53	0.8	28	52.8
**MP13**	7371	1,430	19.4	277	3.8	109	39.4	519	7.0	51	0.7	23	45.1

[1] For assessment of gene/coding regions, more stringent criteria than for assessment of diplotypic transcripts have been used, requiring 95% of the coding sequences to be covered to capture all genes with two or more AA exchanges that could exist in different phase configurations and therefore require phasing.
[2] Predicted by PolyPhen-2[2] and SIFT[3] using default score thresholds of 0.85 and of 0.05, respectively.
[3] Relative to total number of protein-coding sequences containing ≥2 AA exchanges/ ≥2 damaging AA exchanges. Phased protein-coding sequences are entirely contained within phased contigs.

Supplementary Table 6 Molecular versus statistical phasing

A. Phase discordance between molecularly and statistically phased heterozygous SNPs

Subject	No. het SNPs called[1]	No. het SNPs phased[2]	% mol phased	No. het SNPs mol & stat phased[3]	% het SNPs eval[4]	No. discord SNPs[5] global	%[6]	%[7] chr	No. het SNPs eval genes[3]	No. discord SNPs genes	%[6]	%[7] chr	No. het SNPs eval exons[3]	No. discord SNPs exons	%[6]	%[7] chr
**MP2**	1,414,752	1,388,000	98.1	1,306,782	92.4	68,052	5.2	5.9	456,001	21,373	4.7	5.1	9,947	371	3.7	4.2
**MP3**	1,162,750	1,148,010	98.7	1,080,157	92.9	46,645	4.3	4.9	369,136	14,052	3.8	4.1	7,553	250	3.3	3.2
**MP4**	1,128,402	1,113,917	98.7	1,048,192	92.9	41,144	3.9	4.4	372,671	12,749	3.4	3.7	8,090	221	2.7	3.0
**MP5**	1,054,723	1,033,337	98.0	958,846	90.9	42,733	4.5	4.9	337,017	12,781	3.8	4.0	7,637	250	3.3	3.5
**MP6**	1,030,636	993,281	96.4	957,399	92.9	44,837	4.7	5.2	345,428	14,470	4.2	4.5	7,623	253	3.3	3.5
**MP7**	752,602	701,769	93.2	686,408	91.2	39,815	5.8	6.3	251,877	13,553	5.4	5.8	5,403	184	3.4	3.4
**MP8**	712,584	688,560	96.6	657,427	92.3	29,223	4.4	5.0	222,834	8,696	3.9	4.1	4,368	160	3.7	3.8
**MP9**	657,973	592,085	90.0	590,835	89.8	38,001	6.4	7.1	211,008	13,080	6.2	6.7	4,132	183	4.4	4.7
**MP10**	618,033	579,261	93.7	565,710	91.5	28,389	5.0	5.6	191,869	9,210	4.8	5.2	3,632	111	3.1	3.1
**MP11**	601,596	574,718	95.5	558,020	92.8	46,243	8.3	9.3	194,515	15,707	8.1	8.8	3,722	157	4.2	4.4
**MP12**	533,961	475,854	89.1	470,237	88.1	28,848	6.1	6.9	160,671	9,846	6.1	6.7	2,899	112	3.9	3.9
**MP13**	476,169	411,173	86.4	400,633	84.1	19,618	4.9	5.4	141,372	6,755	4.8	5.2	2,970	68	2.3	2.7
**Avg.**	845,348	808,330	94.5	773,387	91.0	39,462	5.3	5.9	271,200	12,689	4.9	5.3	5,665	193	3.4	3.6

[1] Total number of heterozygous SNPs called from the combined sequenced fosmid pools as described in Supplementary Methods.
[2] Number of heterozygous SNPs phased by applying RefHap[4].
[3] Number of heterozygous positions for which both molecular and statistical phase was available for comparative evaluation; equivalent to the numbers of heterozygous SNP positions that were evaluated for phase discordance genome-wide or, where specified, in genes or exons.
[4] Fraction of heterozygous SNPs comparatively evaluated relative to total number of heterozygous SNPs available from fosmid-based molecular data.
[5] Molecular and statistically inferred phase was compared at adjacent SNP pairs using a 'sliding window' approach along phased sequences genome-wide, and the number of phase-discordant SNP positions counted.
[6] Discordance calculated relative to the total (whole genome-based) numbers of heterozygous positions evaluated.
[7] Discordance calculated separately for each of the 22 autosomes, the 'units of phasing', and then averaged.

Strategy: In addition to molecular phase, these 12 genomes were phased statistically using 57CEU datasets from the 1000 Genomes Project database (Pilot Phase)[5] as the required supplementary population data source. Statistical phase was inferred by use of the program fastPhase[6] and comparatively evaluated at the heterozygous positions that were shared by both molecular and statistical phase data. A sliding window approach was used. The phase-discordant SNP positions were counted. See also Supplementary Methods.

B. Phase discordance between molecularly and statistically phased SNPs in disease-related[1] genes

Subject	Fraction/Number of phase-discordant SNPs		
	OMIM (%)	GAD (%)	GWAS[2]
MP2	5.3	5.2	17
MP3	4.4	4.2	11
MP4	4.0	3.7	9
MP5	4.4	4.3	16
MP6	5.0	5.1	10
MP7	6.4	6.1	14
MP8	4.8	4.4	8
MP9	7.6	7.0	19
MP10	6.1	6.2	3
MP11	9.4	9.6	8
MP12	8.1	7.7	6
MP13	6.9	6.6	9
Avg.	6.0	5.8	10.8

[1] GWAS, GAD and OMIM data obtained from UCSC (hg18) table browser.
[2] Absolute numbers of SNPs presented due to their relatively low number.

Supplementary Table 7 Control of selection bias due to sub-sampling

Randomly selected sets of 10 genomes[1]	Unique haplotypes			Unique diplotypes		
	No.	difference[2]	%[3]	No.	difference[2]	%[3]
Random set 1	219,509	0	0	145,165	0	0
Random set 2	219,872	363	0.17	144,490	675	0.46
Random set 3	219,532	23	0.01	144,620	545	0.38
Random set 4	219,703	194	0.09	144,523	642	0.44
Random set 5	219,530	21	0.01	144,715	450	0.31
Random set 6	219,817	308	0.14	144,311	854	0.59
Random set 7	220,011	502	0.23	144,841	324	0.22
Random set 8	219,668	159	0.07	145,023	142	0.10
Random set 9	219,947	438	0.20	144,450	715	0.49
Random set 10	219,830	321	0.15	144,525	640	0.44

[1] Selected from 57CEU from the 1000 Genomes Project database, Pilot Phase (Abecasis et al. 2010)[5], ftp://ftp.1000genomes.ebi.ac.uk/vol1/ftp/release/ 2009_04/).
[2] Difference to 'Random set 1' (reference set).
[3] Difference relative to total number of unique haplotypes/diplotypes assessed in 'Random set 1'.

Supplementary Table 8 Gene haplotypes and diplotypes in European population samples

A. Unique gene haplotypes and diplotypes per total input count[1]

	14G[2] No. genomes			1000G[3] No. genomes				
	**5**	**10**	**14**	**5**	**10**	**14**	**57**	**372**
**Unique haplotypes (%)[4]**	79.50	68.70	63.40	74.32	67.18	62.11	43.67	33.51
**Unique diplotypes (%)[4]**	93.40	86.86	81.81	91.11	87.16	84.40	74.60	63.32

[1] Total input count gene haplotypes: number of phased genes per genome x 2, multiplied by number of genomes assessed; total input count gene diplotypes: number of phased genes per genome multiplied by number of genomes assessed (half of haplotype input count).
[2] Data source: 14 molecularly haplotype-resolved genomes using fosmid pool-based next generation sequencing (14G), including the 12 novel haplotype-resolved genomes described and MP1[4] and NA12878[5]; sets of 5, 10, and 14 thereof were analyzed.
[3] Data source: 1000 Genomes Project database (1000G) providing statistically haplotype-resolved genomes; 57CEU[5] and subsets thereof, 5, 10 and 14 genomes, and the entire set of European ancestry-based genomes, 372EUR[7] were analyzed.
[4] Numbers of unique gene haplotypes/diplotypes divided by total haplotype/diplotype input count.

B. Numbers of unique gene haplotypes and diplotypes and total input counts

	14G No. genomes			1000G No. genomes				
	**5**	**10**	**14**	**5**	**10**	**14**	**57**	**372**
**No. unique haplotypes[1]**	47,208	14,859	3,762	123,313	222,930	288,582	826,069	4,137,353
**No. haplotypes measured[2]**	59,360	21,620	5,936	165,930	331,860	464,604	1,891,602	12,345,192
**No. unique diplotypes[1]**	27,721	10,080	2,428	75,587	144,620	195,966	705,233	3,908,600
**No. diplotypes measured[2]**	29,680	10,810	2,968	82,965	165,930	232,302	945,801	6,172,596

[1] Different, or unique gene haplotypes/diplotypes.
[2] Total input count gene haplotypes: number of phased genes per genome x 2, multiplied by number of genomes assessed; total input count gene diplotypes: number of phased genes per genome multiplied by number of genomes assessed (half of haplotype input count).

C. Average numbers of unique haplotypes and diplotypes 'per gene'

	14G No. genomes			1000G No. genomes				
	**5**	**10**	**14**	**5**	**10**	**14**	**57**	**372**
**Avg. no. haplotypes[1] 'per gene'**	7.95	13.75	17.75	7.43	13.44	17.39	49.78	249.34
**Avg. no. diplotypes[1] 'per gene'**	4.67	8.69	11.45	4.56	8.72	11.81	42.50	235.56

[1] Averages 'per gene' calculated as follows: Total number of unique gene haplotypes/diplotypes per genome x number of genomes divided by number of autosomal genes assessed.

Supplementary Table 9 Extrapolation of numbers of unique haplotypes and diplotypes to larger population samples

No. genomes	No. unique haplotypes[1] (Mio)[3]	(%)[4]	Avg. no. unique haplotypes 'per gene'	No. unique diplotypes[2] (Mio)[3]	(%)[4]	Avg. no. unique diplotypes 'per gene'
**Gene level**						
10,000	57	17	3,448	80	48	4,867
100,000	368	11	22,212	666	40	40,170
1,000,000	2,300	7	143,099	5,500	33	331,535
1,000,000[5]	1,700	5	106,494	4,300	26	263,998
**Protein level**						
10,000	0.3	0.1	21	0.7	0.5	46
100,000	0.8	0.02	48	2	0.1	129
1,000,000	1.8	0.005	110	6	0.04	367

[1] Gene haplotype approximation: $y_a=97.006*x^{-0.1803}$, $R^2=0.96$, protein haplotype approximation: $y_c=38.346*x^{-0.640}$, $R^2=0.9475$ (see Supplementary Methods).

[2] Gene diplotype approximation : $y_b=104.87*x^{-0.0836}$, $R^2=0.99$, protein diplotype approximation: $y_d=70.173*x^{-0.547}$, $R^2=0.9582$.

[3] Values are approximations.

[4] Relative to total haplotype/diplotype counts for indicated number of genomes.

[5] Numbers corrected for potential over-estimation by ~25% of haplotypes due to phasing (switch) errors, as determined by probability analyses in 372EUR (Supplementary Methods).

Supplementary Table 10 Categorization of autosomal genes

A. Fractions of category 1, 2 and 3 genes[1]

	14G No. genomes			1000G No. genomes				
	5	10	14	5	10	14	57	372
**Gene haplotypes**								
Category 1 genes[2] (%)	11.6	13.5	15.1	13.9	13.4	13.5	13.8	13.3
Category 2 genes[3] (%)	45.8	37.2	30.7	53.3	33.0	28.0	35.3	36.4
Category 3 genes[4] (%)	42.7	49.3	54.2	32.8	53.6	58.5	50.9	50.3
**Gene diplotypes**								
Category 1 genes[2] (%)	7.7	6.3	7.1	8.1	5.5	5.6	5.2	6.6
Category 2 genes[3] (%)	7.5	17.5	17.4	12.6	12.6	18.2	14.4	17.9
Category 3 genes[4] (%)	92.3	76.2	75.5	91.9	81.9	76.3	80.4	75.5

[1] Numbers of category 1, 2 and 3 genes as defined below, divided by the total of autosomal RefSeq (hg18) genes assessed (%).
[2] Category 1 genes defined by having one major gene haplotype/diplotype accounting for ≥50% of the measured haplotypes.
[3] Category 2 genes defined by having at least one common gene haplotype/diplotype with a frequency ≥20%.
[4] Category 3 genes defined by having 'un-common' gene haplotypes/diplotypes only with a frequency below 20%.

B. Frequencies of occurrence of gene haplotypes and diplotypes[1] constituting category 1, 2 and 3 genes[2]

	14G No. genomes			1000G No. genomes				
	5	10	14	5	10	14	57	372
**Haplotypes**								
**Category 1 genes[2]**								
Major haplotype (%)	72.9	70.2	69.1	74.6	72.2	71.4	70.3	68.1
Common haplotypes (%)	11.6	10.1	13.4	13.6	10.6	9.2	9.2	8.3
Non-common haplotypes (%)	15.5	19.7	17.5	11.8	17.2	19.4	19.5	23.6
Singleton haplotypes[3] (%)	15.5	9.6	4.8	11.8	7.7	5.8	1.8	1.1
**Category 2 genes[2]**								
Common haplotypes (%)	43.9	42.5	45.2	47.4	45.3	45.4	44.4	44.3
Non-common haplotypes (%)	56.1	57.5	54.8	52.6	54.7	54.6	55.6	55.7
Singleton haplotypes[3] (%)	56.1	38.9	31.9	52.6	31.1	22.2	7.7	0.9
**Category 3 genes[2]**								
Non-common haplotypes (%)	100	100	100	100	100	100	100	100
Singleton haplotypes[3] (%)	100*	86.8	83.6	100	72.4	73.1	46.6	43.7
**Diplotypes**								
**Category 1 genes[2]**								
Major diplotype (%)	70.6	71.3	70.5	73.5	76.7	75.4	72.1	73.3
Common diplotypes (%)	4.5	11.2	10.9	3.5	7.8	5.3	2.3	6.5
Non-common diplotypes (%)	24.9	17.5	18.6	23.0	15.5	19.3	25.6	20.2
Singleton diplotypes[3] (%)	24.9	17.5	10.9	23.0	15.5	12.8	5.5	2.2
**Category 2 genes[2]**								
Common diplotypes (%)	45.6	58.9	54.8	46.5	59.0	41.6	38.7	40.1
Non-common diplotypes (%)	54.4	41.1	45.2	53.5	41.0	58.3	61.3	59.9
Singleton diplotypes[3] (%)	54.4	41.1	31.6	53.5	41.0	42.7	19.4	2.5
**Category 3 genes[2]**								
Non-common diplotypes (%)	100	100	100	100	100	100	100	100
Singleton diplotypes[3] (%)	100	100	90.8	100	100	95.5	84.8	65.5

[1] Frequency of occurrence (%): number of (specified) haplotypes/diplotypes assessed per total haplotype/diplotype count.
[2] Definitions of category 1, 2 and 3 genes see Supplementary Table S10A.
[3] Represent a sub-fraction of the 'non-common' gene haplotypes/diplotypes.

C. Average numbers of gene haplotypes and diplotypes 'per gene' for category 1, 2 and 3 genes[1]

	14G No. genomes			1000G No. genomes				
	5	10	14	5	10	14	57	372
**Haplotypes**								
Category 1 genes[1] (Avg. no. haplotypes 'per gene')	3.0	4.2	4.3	2.7	3.7	4.3	6.7	22.4
Category 2 genes[1] (Avg. no. haplotypes 'per gene')	7.3	10.8	13.0	7.1	9.8	10.9	20.2	74.8
Category 3 genes[1] (Avg. no. haplotypes 'per gene')	10.5	18.6	25.2	10.5	16.2	24.1	71.1	399.2
Global average[2] (Avg. no. haplotypes 'per gene')	8.0	13.8	17.8	7.4	13.4	17.4	49.8	249.3
**Diplotypes**								
Category 1 genes[1] (Avg. no. diplotypes 'per gene')	2.4	3.2	3.5	2.2	2.9	3.4	7.2	16.2
Category 2 genes[1] (Avg. no. diplotypes 'per gene')	3.9	6.1	7.1	3.8	6.1	8.5	18.9	61.8
Category 3 genes[1] (Avg. no. diplotypes 'per gene')	5.0	9.7	13.2	5.0	9.7	13.7	51.4	270.8
Global average[2] (Avg. no. diplotypes 'per gene')	4.7	8.7	11.5	4.6	8.7	11.8	42.5	235.6

[1] Definitions see Supplementary Table S10A. For each of the three gene categories, the numbers of unique gene haplotypes/diplotypes (whole-genome counts) were added up across indicated numbers of haplotype-resolved genomes, and divided by the numbers of autosomal genes contained in each category.

[2] The global average 'per gene' shown above is presented for comparison.

Supplementary Table 11 Protein haplotypes and diplotypes[1]

A. Unique protein haplotypes and diplotypes per total input count[2]

	**14G No. genomes**			**1000G No. genomes**				
	**5**	**10**	**14**	**5**	**10**	**14**	**57**	**372**
Unique protein haplotypes (%)[3,4]	18.5	21.9	18.4	16.4	9.2	6.8	2.0	1.1
Unique protein diplotypes (%)[3,4]	39.2	38.9	36.8	33.4	20.6	16.0	5.6	3.4

[1] Analyses were performed analogous to those described for gene haplotypes, using the subset of nsSNPs that cause AA exchanges; thus, 'protein' haplotypes and diplotypes refer to different protein sequences and pairs thereof.
[2] Total input count protein haplotypes: number of phased protein-coding sequences per genome x 2, multiplied by number of genomes assessed; total input count diplotypes: number of phased protein-coding sequences per genome multiplied by number of genomes assessed (half of protein haplotype input count).
[3] Numbers of unique protein haplotypes/diplotypes divided by total protein haplotype/diplotype input count.
[4] After thorough consideration of all potential sources of error we have come to assume that statistical haplotype inference may underestimate the diversity of unique haplotypes due to an inherent tendency to treat similar haplotypes as identical. Such an effect may primarily become evident for protein haplotypes, due to the much low numbers of nsSNPs. This then results in the lower percentages relative to total haplotype input count.

B. Numbers of unique protein haplotypes and diplotypes

	**14G No. genomes**			**1000G No. genomes**				
	**5**	**10**	**14**	**5**	**10**	**14**	**57**	**372**
No. unique protein haplotypes[1,2]	13,250	4,404	738	27,190	30,354	31,656	37,206	140,251
No. unique protein diplotypes[1,2]	18,078	3,542	753	27,678	34,109	37,193	53,048	206,948

[1] Number of unique protein haplotypes/diplotypes depends on total input count, for the set of 10 and 14 genomes.
[2]The reduction in the numbers in 14G is due to the fact that the numbers of fosmid contigs/gene regions which are simultaneously phased across all genomes, decrease with increasing numbers of individuals (Methods).

C. Average numbers of unique protein haplotypes and diplotypes 'per gene'

	**14G No. genomes**			**1000G No. genomes**				
	**5**	**10**	**14**	**5**	**10**	**14**	**57**	**372**
Avg. no. protein haplotypes[1]	3.4	4.1	4.7	2.2	2.6	2.8	7.1	8.8
Avg. no. protein diplotypes[1]	4.9	4.9	4.8	4.6	6.1	6.8	10.8	12.9

[1] Averages 'per gene' calculated as follows: Total number of protein haplotypes/diplotypes per genome x number of genomes divided by number of autosomal genes assessed; average numbers refer to genes with variable coding sequences.

Supplementary Table 12 Fractions of autosomal protein-coding genes encoding major, common, and non-common haploid/diploid protein forms[1]

	14G No. genomes			1000G No. genomes				
	**5**	**10**	**14**	**5**	**10**	**14**	**57**	**372**
**Protein haplotypes**								
Genes w/ major form[2] (%)	80.9	84.3	88.7	81.7	83.3	84.2	85.7	89.7
Genes w/ common form[3] (%)	18.3	15.2	9.6	18.2	16.4	15.5	13.8	9.7
Genes w/ un-common forms[4] (%)	0.8	0.4	1.5	0.1	0.3	0.3	0.5	0.6
**Protein diplotypes**								
Genes w/ major form[5] (%)	78.4	60.3	53.5	75.0	61.6	62.1	63.3	72.9
Genes w/ common form[6] (%)	14.4	30.3	33.5	20.3	33.9	35.8	33.1	23.3
Genes w/ un-common forms[7] (%)	7.2	9.4	13.2	4.7	4.5	2.1	3.6	3.8

[1] Relative to the total of RefSeq (hg18) genes; analyses were performed analogous to those described for gene haplotypes, using the subset of nsSNPs that cause AA exchanges; thus, 'protein haplotypes' refer to different (haploid) protein sequences and 'protein diplotypes' represent pairs thereof.
[2] Genes that have one major/predominant protein haplotype accounting for ≥50% of the measured protein haplotypes.
[3] Genes that have at least one common protein haplotype with a frequency ≥20%.
[4] Genes that have 'un-common' protein haplotypes only with a frequency below 20%.
[5] Genes that have one major/predominant protein diplotype, accounting for ≥50% of the measured diplotypes.
[6] Genes that have at least one common protein diplotype with a frequency ≥20%.
[7] Genes that have 'un-common' protein diplotypes with a frequency below 20%.

Supplementary Table 13 Personal diplotype signatures at the gene and protein level

	14G<br>No. genomes	1000G<br>No. genomes	
	**14**	**57**	**372**
Avg. no. private gene dips	872 (80.5%)[2]	11,916 (71.8%)[2]	9,329 (56.2%)[2]
Avg. no. private prot dips[1]	169 (27.2%)[3]	256 (7.9%)[3]	277 (8.6%)[3]

[1] Prot dips, protein diplotypes.
[2] Relative to all gene diplotypes measured per genome.
[3] Relative to all protein diplotypes measured per genome.

Supplementary Table 14 57CEU population data[1]: Overview of diplotypes at the gene and protein level

	Sample ID	No. het SNPs	No. genes ≥1 het SNP	%[2]	No. genes ≥2 het SNP	%[2]	No. nsSNPs	%[3]	No. genes ≥1 nsSNP	%[2]	No. genes ≥2 nsSNP	%[2]	No. genes *cis*	%[4]	No. genes *trans*	%[4]	No. damaging mutations[5]	%[3]	No. genes ≥ 1 damaging mutations	%[2]	Genes >2 damaging mutations	%[2]	No. genes dam mut *cis*	%[6]	No. genes dam mut *trans*	%[6]
1	**NA06985**	2,123,789	15,148	84.8	13,618	76.2	4,421	0.21	2,883	16.1	867	4.9	521	60.1	346	39.9	1,936	0.09	1,439	8.1	272	1.5	170	62.5	102	37.5
2	**NA06986**	2,234,527	15,352	86.0	14,029	78.5	4,645	0.21	2,972	16.6	938	5.3	548	58.4	390	41.6	2,052	0.09	1,542	8.6	315	1.8	199	63.2	116	36.8
3	**NA06994**	2,157,139	15,067	84.4	13,550	75.9	4,513	0.21	2,895	16.2	857	4.8	524	61.1	333	38.9	1,965	0.09	1,461	8.2	272	1.5	169	62.1	103	37.9
4	**NA07000**	2,163,960	15,142	84.8	13,723	76.8	4,527	0.21	2,919	16.3	872	4.9	509	58.4	363	41.6	1,945	0.09	1,451	8.1	284	1.6	184	64.8	100	35.2
5	**NA07037**	2,181,294	15,381	86.1	13,927	78.0	5,076	0.23	3,237	18.1	986	5.5	578	58.6	408	41.4	2,357	0.11	1,767	9.9	321	1.8	195	60.7	126	39.3
6	**NA07051**	2,233,798	15,386	86.1	13,973	78.2	4,912	0.22	3,236	18.1	913	5.1	529	57.9	384	42.1	2,277	0.10	1,759	9.8	297	1.7	174	58.6	123	41.4
7	**NA07346**	2,148,734	15,087	84.5	13,647	76.4	4,467	0.21	2,951	16.5	878	4.9	527	60.0	351	40.0	2,001	0.09	1,551	8.7	298	1.7	170	57.0	128	43.0
8	**NA07347**	2,115,376	15,073	84.4	13,626	76.3	4,535	0.21	2,959	16.6	889	5.0	514	57.8	375	42.2	2,002	0.09	1,537	8.6	283	1.6	166	58.7	117	41.3
9	**NA07357**	2,174,444	15,215	85.2	13,809	77.3	4,748	0.22	3,122	17.5	921	5.2	552	59.9	369	40.1	2,076	0.10	1,586	8.9	293	1.6	168	57.3	125	42.7
11	**NA11829**	2,152,727	15,249	85.4	13,778	77.1	4,645	0.22	3,022	16.9	888	5.0	526	59.2	362	40.8	2,050	0.10	1,523	8.5	264	1.5	160	60.6	104	39.4
12	**NA11830**	2,179,095	15,160	84.9	13,786	77.2	4,544	0.21	2,968	16.6	899	5.0	543	60.4	356	39.6	2,035	0.09	1,551	8.7	302	1.7	174	57.6	128	42.4
13	**NA11831**	2,110,778	15,030	84.1	13,514	75.7	4,497	0.21	2,912	16.3	864	4.8	516	59.7	348	40.3	2,019	0.10	1,524	8.5	301	1.7	185	61.5	116	38.5
14	**NA11832**	2,099,353	15,100	84.5	13,601	76.1	4,493	0.21	2,942	16.5	859	4.8	529	61.6	330	38.4	1,963	0.09	1,504	8.4	261	1.5	161	61.7	100	38.3
15	**NA11840**	2,088,720	14,989	83.9	13,514	75.7	4,379	0.21	2,865	16.0	879	4.9	514	58.5	365	41.5	1,897	0.09	1,452	8.1	276	1.5	160	58.0	116	42.0
16	**NA11881**	2,115,347	15,030	84.1	13,534	75.8	4,283	0.20	2,840	15.9	810	4.5	505	62.3	305	37.7	1,886	0.09	1,444	8.1	258	1.4	172	66.7	86	33.3
17	**NA11894**	2,208,323	15,380	86.1	13,930	78.0	4,770	0.22	3,152	17.6	928	5.2	525	56.6	403	43.4	2,137	0.10	1,644	9.2	298	1.7	176	59.1	122	40.9
18	**NA11918**	2,207,699	15,249	85.4	13,892	77.8	4,741	0.21	3,033	17.0	912	5.1	505	55.4	407	44.6	2,072	0.09	1,593	8.9	288	1.6	174	60.4	114	39.6
19	**NA11919**	2,253,111	15,385	86.1	14,018	78.5	4,780	0.21	3,085	17.3	919	5.1	517	56.3	402	43.7	2,150	0.10	1,620	9.1	310	1.7	185	59.7	125	40.3
20	**NA11920**	2,235,341	15,299	85.7	13,948	78.1	4,684	0.21	3,046	17.1	899	5.0	531	59.1	368	40.9	2,088	0.09	1,535	8.6	316	1.8	196	62.0	120	38.0
21	**NA11931**	2,217,622	15,286	85.6	13,891	77.8	4,890	0.22	3,159	17.7	972	5.4	582	59.9	390	40.1	2,145	0.10	1,623	9.1	311	1.7	199	64.0	112	36.0
22	**NA11992**	2,223,418	15,338	85.9	13,896	77.8	4,592	0.21	2,990	16.7	855	4.8	481	56.3	374	43.7	1,974	0.09	1,493	8.4	261	1.5	147	56.3	114	43.7
23	**NA11993**	2,167,468	15,158	84.9	13,735	76.9	4,588	0.21	2,998	16.8	891	5.0	507	56.9	384	43.1	2,042	0.09	1,556	8.7	275	1.5	161	58.5	114	41.5
24	**NA11994**	2,360,317	15,561	87.1	14,262	79.8	5,898	0.25	3,692	20.7	1262	7.1	704	55.8	558	44.2	3,001	0.13	2,184	12.2	514	2.9	303	58.9	211	41.1
25	**NA11995**	2,159,131	15,222	85.2	13,781	77.2	4,569	0.21	3,042	17.0	890	5.0	526	59.1	364	40.9	2,034	0.09	1,563	8.8	285	1.6	174	61.1	111	38.9
26	**NA12003**	2,219,934	15,293	85.6	13,875	77.7	4,476	0.20	2,949	16.5	850	4.8	504	59.3	346	40.7	1,958	0.09	1,499	8.4	261	1.5	162	62.1	99	37.9
27	**NA12005**	2,142,763	14,996	84.0	13,605	76.2	4,422	0.21	2,843	15.9	861	4.8	485	56.3	376	43.7	2,009	0.09	1,489	8.3	314	1.8	200	63.7	114	36.3
28	**NA12006**	2,174,665	15,220	85.2	13,740	76.9	4,655	0.21	3,016	16.9	892	5.0	520	58.3	372	41.7	2,038	0.09	1,540	8.6	294	1.6	178	60.5	116	39.5

29	**NA12043**	2,148,946	15,194	85.1	13,785	77.2	4,606	0.21	2,979	16.7	887	5.0	523	59.0	364	41.0	2,164	0.10	1,609	9.0	316	1.8	194	61.4	122	38.6
30	**NA12044**	2,207,444	15,322	85.8	13,866	77.6	4,754	0.22	3,115	17.4	906	5.1	506	55.8	400	44.2	2,183	0.10	1,671	9.4	315	1.8	171	54.3	144	45.7
31	**NA12045**	2,111,342	15,066	84.4	13,605	76.2	4,543	0.22	2,955	16.5	888	5.0	529	59.6	359	40.4	2,010	0.10	1,522	8.5	284	1.6	180	63.4	104	36.6
32	**NA12144**	2,195,915	15,112	84.6	13,709	76.8	4,530	0.21	2,924	16.4	875	4.9	498	56.9	377	43.1	1,902	0.09	1,426	8.0	258	1.4	162	62.8	96	37.2
33	**NA12154**	2,145,302	15,127	84.7	13,698	76.7	4,548	0.21	2,930	16.4	912	5.1	525	57.6	387	42.4	2,018	0.09	1,519	8.5	300	1.7	185	61.7	115	38.3
34	**NA12155**	2,171,593	15,314	85.7	13,850	77.5	4,753	0.22	3,011	16.9	883	4.9	494	55.9	389	44.1	2,116	0.10	1,579	8.8	294	1.6	180	61.2	114	38.8
35	**NA12156**	2,168,133	15,216	85.2	13,686	76.6	4,721	0.22	2,996	16.8	938	5.3	529	56.4	409	43.6	2,068	0.10	1,561	8.7	291	1.6	180	61.9	111	38.1
36	**NA12234**	2,213,026	15,042	84.2	13,586	76.1	4,635	0.21	2,976	16.7	898	5.0	501	55.8	397	44.2	2,090	0.09	1,559	8.7	291	1.6	187	64.3	104	35.7
37	**NA12249**	2,160,725	15,111	84.6	13,702	76.7	4,757	0.22	2,986	16.7	880	4.9	501	56.9	379	43.1	2,157	0.10	1,595	8.9	285	1.6	175	61.4	110	38.6
38	**NA12287**	2,212,614	15,354	86	13,958	78.1	4,761	0.22	3,127	17.5	941	5.3	546	58.0	395	42.0	2,118	0.10	1,632	9.1	310	1.7	187	60.3	123	39.7
39	**NA12489**	2,169,402	15,260	85.4	13,922	77.9	4,804	0.22	3,074	17.2	955	5.3	537	56.2	418	43.8	2,101	0.10	1,606	9.0	291	1.6	169	58.1	122	41.9
40	**NA12716**	2,254,057	15,468	86.6	14,129	79.1	4,949	0.22	3,287	18.4	980	5.5	563	57.4	417	42.6	2,349	0.10	1,794	10.0	343	1.9	208	60.6	135	39.4
41	**NA12749**	2,263,648	15,380	86.1	14,057	78.7	6,445	0.28	3,663	20.5	1355	7.6	717	52.9	638	47.1	3,429	0.15	2,222	12.4	615	3.4	338	55.0	277	45.0
42	**NA12750**	2,242,263	15,413	86.3	14,041	78.6	5,098	0.23	3,301	18.5	978	5.5	564	57.7	414	42.3	2,302	0.10	1,771	9.9	327	1.8	201	61.5	126	38.5
43	**NA12751**	2,288,412	15,494	86.7	14,104	79.0	5,092	0.22	3,313	18.5	949	5.3	548	57.7	401	42.3	2,385	0.10	1,778	10.0	315	1.8	189	60.0	126	40.0
44	**NA12761**	2,262,420	15,438	86.4	14,075	78.8	4,900	0.22	3,176	17.8	951	5.3	553	58.1	398	41.9	2,281	0.10	1,741	9.7	326	1.8	207	63.5	119	36.5
45	**NA12763**	2,156,404	15,249	85.4	13,861	77.6	4,841	0.22	3,097	17.3	942	5.3	552	58.6	390	41.4	2,167	0.10	1,636	9.2	297	1.7	183	61.6	114	38.4
46	**NA12776**	2,204,312	15,288	85.6	13,882	77.7	4,855	0.22	3,136	17.6	948	5.3	555	58.5	393	41.5	2,234	0.10	1,704	9.5	317	1.8	183	57.7	134	42.3
47	**NA12812**	2,202,296	15,281	85.6	13,878	77.7	4,887	0.22	3,101	17.4	956	5.4	565	59.1	391	40.9	2,187	0.10	1,642	9.2	328	1.8	185	56.4	143	43.6
48	**NA12813**	2,147,772	15,235	85.3	13,779	77.1	4,676	0.22	3,033	17.0	885	5.0	525	59.3	360	40.7	2,063	0.10	1,550	8.7	306	1.7	197	64.4	109	35.6
49	**NA12814**	2,213,692	15,255	85.4	13,830	77.4	4,884	0.22	3,128	17.5	945	5.3	539	57.0	406	43.0	2,264	0.10	1,684	9.4	311	1.7	176	56.6	135	43.4
50	**NA12815**	2,269,902	15,133	84.7	13,689	76.6	4,652	0.20	3,009	16.8	896	5.0	545	60.8	351	39.2	2,083	0.09	1,573	8.8	307	1.7	186	60.6	121	39.4
51	**NA12828**	2,201,064	15,374	86.1	13,945	78.1	4,960	0.23	3,140	17.6	973	5.4	554	56.9	419	43.1	2,284	0.10	1,727	9.7	358	2.0	218	60.9	140	39.1
52	**NA12872**	2,224,547	15,318	85.8	13,923	78.0	4,944	0.22	3,134	17.5	929	5.2	507	54.6	422	45.4	2,227	0.10	1,632	9.1	314	1.8	175	55.7	139	44.3
53	**NA12873**	2,113,992	15,035	84.2	13,537	75.8	4,482	0.21	2,941	16.5	844	4.7	495	58.6	349	41.4	2,056	0.10	1,549	8.7	304	1.7	180	59.2	124	40.8
54	**NA12874**	2,131,049	14,652	82.0	13,267	74.3	4,734	0.22	2,975	16.7	917	5.1	499	54.4	418	45.6	2,105	0.10	1,573	8.8	290	1.6	162	55.9	128	44.1
56	**NA12891**	2,119,488	15,094	84.5	13,623	76.3	4,512	0.21	2,878	16.1	860	4.8	504	58.6	356	41.4	1,940	0.09	1,456	8.2	280	1.6	188	67.1	92	32.9
57	**NA12892**	2,126,144	15,055	84.3	13,512	75.7	4,528	0.21	2,945	16.5	890	5.0	539	60.6	351	39.4	1,989	0.09	1,510	8.5	278	1.6	188	67.6	90	32.4
	**Avg.**	**2,184,996**	**15,220**	**85.2**	**13,795**	**77.2**	**4,738**	**0.22**	**3,056**	**17.1**	**920**	**5.2**	**533**	**58.0**	**387**	**42.0**	**2,134**	**0.1**	**1,605**	**9.0**	**307**	**1.7**	**185**	**60.6**	**121**	**39.4**

1 European ancestry-based 57CEU samples from the 1000 Genomes Project database, Pilot Phase[5]; ftp://ftp.1000genomes.ebi.ac.uk/vol1/ftp/release/ 2009_04/)
2 Relative to total number of autosomal RefSeq (hg18) genes.
3 Relative to total number of heterozygous SNPs.
4 Relative to number of genes with ≥2 nsSNPS.
5 Predicted by PolyPhen-2[2] and SIFT[3].
6 Relative to number of genes with ≥2 damaging mutations.

Supplementary Table 15 Subsets of autosomal genes encoding protein diplotypes in European population samples

A. Number of genes exhibiting protein diplotypes above defined frequency thresholds

	Number of genes exhibiting protein diplotypes[1]							
**Frequency thresholds[2]**	**14G**	**%[5]**	**14-57CEU[7]**	**%[5]**	**57CEU**	**%[5]**	**372EUR**	**%[5]**
**1 genome (singleton)[3]**	2,376	13.3	1,754	9.8	1,118	6.3	1,161	6.5
**≥1 genome**	8,587	48.1	8,631	48.3	9,727	54.5	15,903	89.0
**≥2 genomes**	6,211	34.8	6,877	38.5	8,609	48.2	14,742	82.5
**≥5 %[4]**	8,587	48.1[6]	8,631	48.3[6]	7,989	44.7	9,920	55.5
**≥20 %**	4,934	27.6	5,921	33.1	5,839	32.7	7,117	39.8
**≥30 %**	2,913	16.3	4,405	24.7	4,665	26.1	5,951	33.3
**≥50 %**	1,530	8.6	2,976	16.6	2,698	15.1	3,324	18.6
**≥70 %**	377	2.1	1,034	5.8	753	4.2	941	5.3
**≥90 %**	57	0.3	151	0.8	90	0.5	109	0.6

[1] Defined by presence of at least one nsSNP.
[2] Defined by number or fraction of genomes (relative to total genome count), where the gene exhibits a protein diplotype.
[3] Number of genes encoding a protein diplotype in only one genome, 'singleton protein diplotype'.
[4] Relative to total genome count.
[5] Relative to the total of autosomal RefSeq (hg18) genes.
[6] Data refer to one genome, equivalent to 5% of total genome count in the 14 molecularly haplotype-resolved genomes.
[7] Subset of 14 genomes selected from 57CEU, 1000 Genomes Project database (Pilot Phase)[5], for control of selection bias see Supplementary Methods

B. Intersection of gene sets encoding protein diplotypes above defined frequency thresholds

	Number of genes encoding protein diplotypes			Genes in overlap 14G ∩ 57CEU (Set I)		Genes in overlap 57CEU ∩ 372EUR[4] (Set II)		Merged Sets I and II[3]
**Frequency threshold[1]**	**14G**	**57CEU**	**372EUR**	**No. genes**	**%[2]**	**No. genes**	**%[2]**	**No. genes**
≥5 %	8,587	7,989	9,920	6,420	80.4	7,027	88.0	6328
≥20 %	4,934	5,839	7,117	4,038	81.8	5,220	89.4	5163
≥30 %	2,913	4,665	5,951	2,412	82.8	4,204	90.1	4269
≥50 %	1,530	2,486	3,324	1,038	67.8	2,025	81.5	2102
≥70 %	377	753	941	202	53.6	472	62.7	511
≥90 %	57	63	109	18	31.6	31	49.2	35

[1] Fraction of genomes (relative to total genome count), where the gene exhibits a protein diplotype.
[2] Fractions of genes in overlap calculated relative to the smaller sample set.
[3] Each (unique) diplotypic gene in the final, merged gene set is present in at least two of three distinct sample sets (see also Supplementary Methods).

Supplementary Table 16 Whole genome *cis*-abundance of potentially perturbing mutations in 14 molecularly haplotype-resolved genomes

Subject	Total[1]	*Cis*[2]	%[3]	*Trans*[4]	%[3]
**MP1**	258	147	57.0	111	43.0
**NA12878**	202	112	55.4	90	44.6
**MP2**	175	114	65.1	61	34.9
**MP3**	125	81	64.8	44	35.2
**MP4**	127	72	56.7	55	43.3
**MP5**	125	81	64.8	44	35.2
**MP6**	116	75	64.7	41	35.3
**MP7**	81	52	64.2	29	35.8
**MP8**	52	38	73.1	14	26.9
**MP9**	41	30	73.2	11	26.8
**MP10**	31	19	61.3	12	38.7
**MP11**	43	31	72.1	12	27.9
**MP12**	28	18	64.3	10	35.7
**MP13**	23	14	60.9	9	39.1
**Avg.**	102	63	64.1	39	35.9

[1] Total number of autosomal protein-coding genes with potentially perturbing mutations predicted by PolyPhen-2[2] and SIFT[3].
[2] Number of genes with mutations residing on the same chromosome, in '*cis* configurations'.
[3] Relative to total number of genes with potentially perturbing mutations.
[4] Number of genes with mutations residing on opposite chromosomes, in '*trans* configurations'.

Supplementary Table 17 Dissection of *cis* and *trans* configurations in relation to the numbers of mutations

A. *Cis* and *trans* configurations of potentially damaging mutations

1000G-57CEU							14G						
**No. damaging mutations**	**No. configs**	**%[1]**	**No. configs *cis*[2]**	**No. configs *trans*[3]**	**% *cis***	**% *trans***	**No. damaging mutations**	**No. configs**	**%[1]**	**No. configs *cis*[2]**	**No. configs *trans*[3]**	**% *cis***	**% *trans***
**2**	12,067	71.51	8,102	3,965	67.14	32.86	**2**	1,302	73.98	872	430	66.97	33.03
**3**	3,017	17.88	1,508	1,509	49.98	50.02	**3**	278	15.80	144	134	51.80	48.20
**4**	827	4.90	341	486	41.23	58.77	**4**	77	4.38	37	40	48.05	51.95
**5**	381	2.26	119	262	31.23	68.77	**5**	32	1.82	9	23	28.13	71.88
**6**	234	1.39	68	166	29.06	70.94	**6**	28	1.59	15	13	53.57	46.43
**7**	127	0.75	34	93	26.77	73.23	**7**	12	0.68	3	9	25.00	75.00
**8**	72	0.43	7	65	9.72	90.28	**8**	9	0.51	3	6	33.33	66.67
**9**	67	0.40	4	63	5.97	94.03	**9**	5	0.28	1	4	20.00	80.00
**10**	28	0.17	3	25	10.71	89.29	**10**	2	0.11	0	2	0.00	100.00
**11**	8	0.05	1	7	12.50	87.50	**11**	4	0.23	0	4	0.00	100.00
**12**	12	0.07	4	8	33.33	66.67	**12**	2	0.11	0	2	0.00	100.00
**13**	6	0.04	1	5	16.67	83.33	**13**	2	0.11	0	2	0.00	100.00
**14**	6	0.04	3	3	50.00	50.00	**15**	1	0.06	0	1	0.00	100.00
**15**	3	0.02	1	2	33.33	66.67	**16**	1	0.06	0	1	0.00	100.00
**16**	2	0.01	0	2	0.00	100.00	**17**	1	0.06	0	1	0.00	100.00
**17**	4	0.02	0	4	0.00	100.00	**18**	2	0.11	0	2	0.00	100.00
**18**	4	0.02	0	4	0.00	100.00	**19**	0	0.00	0	0	na	na
**19**	3	0.02	0	3	0.00	100.00	**20**	0	0.00	0	0	na	na
**20**	3	0.02	0	3	0.00	100.00	**21**	1	0.06	0	1	0.00	100.00
**21**	3	0.02	0	3	0.00	100.00	**22**	1	0.06	0	1	0.00	100.00
**23**	1	0.01	0	1	0.00	100.00	**23**	0	0.00	0	0	na	na
							**24**	1	0.06	0	1	na	na
**Total**	16,875	100	10,196	6,679			**Total**	1,761	100	1,084	676		
**Avg.**					60.42	39.58	**Avg.**					61.56	38.39

[1] Fractions relative to total number of configurations.
[2] Number of *cis* configurations of potentially damaging mutations.
[3] Number of *trans* configurations of potentially damaging mutations.

Supplementary Table 17 Dissection of *cis* and *trans* configurations in relation to the numbers of mutations in genes

B. *Cis* and *trans* configurations of AA exchanges

1000G-57CEU							14G						
**No. AA exchanges**	**No. configs**	**%[1]**	**No. configs *cis*[2]**	**No. configs *trans*[3]**	**% *cis***	**% *trans***	**No. AA exchanges**	**No. configs**	**%[1]**	**No. configs *cis*[2]**	**No. configs *trans*[3]**	**% *cis***	**% *trans***
**2**	31,162	61.57	20,940	10,222	67.20	32.80	**2**	4,231	64.75	2832	1399	66.93	33.07
**3**	10,421	20.59	5,263	5,158	50.50	49.50	**3**	1,281	19.61	674	607	52.62	47.38
**4**	4,153	8.21	1,761	2,392	42.40	57.60	**4**	481	7.36	246	235	51.14	48.86
**5**	1,968	3.89	716	1,252	36.38	63.62	**5**	187	2.86	79	108	42.25	57.75
**6**	950	1.88	338	612	35.58	64.42	**6**	114	1.74	35	79	30.70	69.30
**7**	454	0.90	70	384	15.42	84.58	**7**	60	0.92	12	48	20.00	80.00
**8**	362	0.72	120	242	33.15	66.85	**8**	46	0.70	3	43	6.52	93.48
**9**	238	0.47	48	190	20.17	79.83	**9**	31	0.47	5	26	16.13	83.87
**10**	168	0.33	41	127	24.40	75.60	**10**	20	0.31	2	18	10.00	90.00
**11**	124	0.25	21	103	16.94	83.06	**11**	14	0.21	2	12	14.29	85.71
**12**	93	0.18	3	90	3.23	96.77	**12**	14	0.21	2	12	14.29	85.71
**13**	79	0.16	2	77	2.53	97.47	**13**	9	0.14	0	9	0.00	100.00
**14**	73	0.14	1	72	1.37	98.63	**14**	6	0.09	0	6	0.00	100.00
**15**	81	0.16	3	78	3.70	96.30	**15**	3	0.05	0	3	0.00	100.00
**16**	44	0.09	2	42	4.55	95.45	**16**	8	0.12	0	8	0.00	100.00
**17**	43	0.08	3	40	6.98	93.02	**17**	4	0.06	0	4	0.00	100.00
**18**	50	0.10	1	49	2.00	98.00	**18**	4	0.06	1	3	25.00	75.00
**19**	21	0.04	1	20	4.76	95.24	**19**	2	0.03	0	2	0.00	100.00
**20**	18	0.04	1	17	5.56	94.44	**20**	2	0.03	0	2	0.00	100.00
**21**	26	0.05	0	26	0.00	100.00	**21**	1	0.02	0	1	0.00	100.00
**22**	18	0.04	0	18	0.00	100.00	**22**	2	0.03	0	2	0.00	100.00
**23**	10	0.02	0	10	0.00	100.00	**23**	1	0.02	0	1	0.00	100.00
**24**	14	0.03	0	14	0.00	100.00	**24**	2	0.03	1	1	50.00	50.00
**25**	6	0.01	0	6	0.00	100.00	**25**	1	0.02	0	1	0.00	100.00
**26**	7	0.01	0	7	0.00	100.00	**26**	1	0.02	0	1	0.00	100.00
**27**	7	0.01	0	7	0.00	100.00	**27**	1	0.02	0	1	0.00	100.00
**28**	3	0.01	0	3	0.00	100.00	**30**	3	0.05	0	3	0.00	100.00
**29**	9	0.02	0	9	0.00	100.00	**32**	1	0.02	0	1	0.00	100.00
**32**	1	0.00	0	1	0.00	100.00	**33**	1	0.02	0	1	0.00	100.00
**33**	2	0.00	0	2	0.00	100.00	**41**	1	0.02	0	1	0.00	100.00
**34**	1	0.00	0	1	0.00	100.00	**44**	1	0.02	0	1	0.00	100.00
**35**	2	0.00	0	2	0.00	100.00	**47**	1	0.02	0	1	0.00	100.00
**37**	1	0.00	0	1	0.00	100.00	**50**				1		
**38**	1	0.00	0	1	0.00	100.00							
**47**	2	0.00	0	2	0.00	100.00							
**Total**	50,612	100	29,335	21,277			**Total**	6,534	100	3894	2641		
**Avg.**					57.96	42.04	**Avg.**					59.60	40.42

[1] Fractions relative to total number of configurations.
[2] Number of *cis* configurations of AA exchanges.
[3] Number of *trans* configurations of AA exchanges; configurations were evaluated for the minor alleles.

Supplementary Notes

Supplementary Note 1

Molecular vs statistical phasing data

Statistical phase information was available at an average of 91% of the molecularly phased heterozygous SNP positions (~400,000–1.3 Mio), which could therefore be comparatively evaluated (Table S6a). Of these positions, 5.9% on average (4.4 - 9.3%) were found to be discordant across all chromosomes (Supplementary Table 6a). This is in very good agreement with the phase discordance obtained for MP1 (6.4%), which was virtually completely haplotype-resolved[4]. Discrepancies to statistical phasing were higher in regions containing rare and novel SNPs, as also described earlier4. Focusing on the subsets of heterozygous SNP positions residing within genes/transcripts, the average phase discordance was slightly lower, 5.3%, and decreased to 3.6% when evaluating exonic sequences (Supplementary Table 6a). The high accuracy of our fosmid-based phasing approach has recently been confirmed by us, when comprehensively haplotype-resolving a HapMap trio child, NA128785. Notably, of the fractions of statistically phased SNPs that were found discordant with molecular phase, roughly equal portions of 6% on average were located in disease genes (OMIM) and in the Genome Association Database (GAD), and 11 discordant SNPs per individual on average corresponded to GWA signals (Table S6b). Wrong attribution to haplotype background of these SNPs may severely hamper processes of disease gene identification.

Supplementary Note 2

Analysis of gene haplotype and diplotype diversity

The entirety/diversity of unique gene haplotypes and diplotypes was determined separately for sample sets of 5, 10 and 14 molecularly haplotype-resolved genomes, corresponding subsets of 5, 10 and 14 statistically resolved genomes extracted from 57CEU, the total set of 57CEU, and 372EUR. Once the lists of unique gene haplotypes and diplotypes for each of these defined sample sets were generated (Methods), gene haplotype and diplotype diversity was analyzed and described in three aspects: 1) as fractions of unique gene haplotypes per total haplotype input count, defined as the number of phased genes (transcripts) per genome x 2, multiplied by the number of genomes assessed; as fractions of unique gene diplotypes, total input count defined as the number of phased genes (transcripts) per genome multiplied by the number of genomes assessed (half of haplotype input count!); 2) in absolute numbers adding whole genome counts of unique gene haplotypes/diplotypes across defined numbers of genomes; 3) as global averages ‘per gene’, calculated as the total number of unique gene haplotypes/diplotypes per genome x the number of genomes divided by the number of autosomal genes assessed.

Supplementary Note 3

Relationships between gene categories, gene length and GO enrichment

To corroborate against potential bias in enrichment analyses, we have performed additional analyses showing that the relationship between gene length and enriched GO categories[8] does not appear to exist so straightforwardly in our data. While on average, category 1 genes are indeed shorter than category 3 genes, we find GO groups related to nervous system functions and disease in category 1 as well as in category 3 genes. Addressing “genes that

buck the trend between diversity and gene length", we analyzed moreover GO and disease enrichment among the 200 longest category 1 genes (12–130 kb) and 200 shortest category 3 genes (1–8 kb). We still find brain diseases enriched among long category 1 genes. Altogether, this indicates that genes of the nervous system can be within category 1 and 3, and are not merely enriched because they are longer. Inspecting the "genes that buck the trend between diversity and gene length" more closely, we find that long genes within category 1 had either very low numbers of SNPs, such as for example the *CBWD5* gene (7 SNPs within 48 kb), or multiple rare SNPs (e.g. 179 SNPs in *LRRC37A3*, 64 kb, or 348 SNPs *BUB1,* in 40 kb), both scenarios giving rise to major haplotypes. Inspecting the short genes within category 3, genes showing a high SNP density within few kb, such as for example the *HLA-C* gene (238 SNPs within 3 kb), or the *TMEM88B* or *ATP6V1G2* genes, showing multiple (33) common SNPs within ~2.3 kb, can lead to a multiplicity of un-common haplotypes. Thus, differences in diversity/haplotype spectra appear to be the result of a more complex relationship between gene length, and the numbers and frequencies of SNPs.

At last, even if the relationship between diversity and gene length would be straightforward, normalizing input sets for GO analyses for gene length does not seem indicated. Young et al., Genome Biology, 2010[8], had originally developed GOseq to correct for experimental/methodological bias in differential expression data introduced by RNAseq due to over-detection of differential expression for long and highly expressed transcripts. (Thus, their work involves GO ranks in relation to transcript length and read counts; however, does not consider SNPs and their variability at all.) An inherent key assumption is that "longer genes are not of biologically greater interest than shorter genes, *per se*". Several lines of evidence suggest, however, that gene length matters (http://sfari.org/news-and-opinion/viewpoint/2013/length-matters-disease-implications-for-long-genes), for instance significantly influencing transcription and splicing mechanisms. But also the biology of (very) short genes can be different from that of average-sized genes (http://mbg.au.dk/en/news-and-events/news-item/artikel/length-matters-in-gene-expression/). The exon/intron architecture of genes, intron size, may profoundly affect splicing mechanisms[9]. Bigger genes are bigger targets, and longer genes are more likely to be hit by random mutations than shorter genes. That is, they have a higher probability of being functionally diverse and dysfunctional. Notably, relatively more diseases have been found enriched in our category 3 genes.

Supplementary Note 4

***Cis*/*trans* ratio in relation to gene categories**

We have also assessed *cis* and *trans* configurations separately for category 2 and 3 genes (excluding category 1 genes) to test for a potential artificial excess of *cis* mutations at genes in which a single main haplotype predominated. The *cis*/*trans* ratio of mutations remained significant in category 2 and 3 genes, with nearly the same ratio (60.3/39.7%) as compared to 61.7/38.3% obtained from all three gene categories, as described in main text.

Supplementary Note 5

***Cis*/*trans* ratio in relation to inter-mutation distances**

Genomic *cis*-abundance is mainly driven by pairs of mutations that are overwhelmingly in a *cis* configuration. To further elucidate this finding, we examined the relationship of configurations with inter-mutation distance and mutation frequency. To this end, we assessed, firstly, all pairs of mutations in the largest population sample 372EUR genomes, evaluating for all *cis* vs all *trans* configurations their median inter-mutation distances. As assumed, the pairs of mutations in *cis* were found to be more closely spaced than the pairs

of mutations in *trans*, at distances of 1.2 kb vs 4.4 kb, respectively. Specifically, 67.4% of all pairs of mutations were in *cis*, and 32.6% in *trans*, virtually identical with the results shown for the 14 molecularly resolved genomes (66.9%/33.1%), and 57CEU (67.2%/32.8%). Secondly, we examined the *cis*/*trans* ratio in relation to increasing inter-mutation genome distances. To this end, we sorted in a first step the *cis* and *trans* configurations by inter-mutation distance. Then we binned per 10,000 *cis* or *trans* observations and calculated for each bin the *cis*/*trans* ratio and the average genomic distance in bp (5' – 3'). For average (genomic) distances between 20 and 1,182 bp, the relative fractions of *cis* configurations were between 82 and 69%. Up to an inter-mutation distance of 27,446 bp, *cis* configurations were still in excess of 60%. The remaining 10% of mutation pairs were in *cis* in at least 50% of the cases, up to an inter-mutation distance of 93,765 bp. Notably, ~28% of all pairs of mutations in *cis* were found to exist within an interval of 250 bp (Tables S4a-c).

Supplementary Note 6

Cis/*trans* ratio in relation to mutation frequencies

Subsequently we examined the influence of mutation frequencies on the *cis*/*trans* ratio. For this we compared pairs of common mutations, the top 10% of configurations at an average frequency of 0.23, with pairs of rare mutations, the bottom 10% (average frequency 0.0037). (Frequencies were defined by number of mutations per total allele count in 372 genomes.) The pairs of common mutations were found to reside in *cis* in 84.3% of the cases, and the pairs of rare mutations existed in *trans* in 50.6% of the cases.

Supplementary Note 7

Analysis of phase differences

In principle, phase differences between genomes and genes can only be identified where heterozygous sites are shared. They refer to the situation, where two SNPs, or mutations, reside on the same chromosome in one genome, and on opposite chromosomes in the other genome. Because the fractions of shared heterozygous sites decrease rapidly when intersecting multiple genomes, with roughly 2% shared between 7 genomes and approaching zero between 14 genomes (data not shown), our analyses were based on the identification of phase differences between pairs of genomes. Furthermore, they were performed at the protein level to extract pairs of potentially perturbing mutations that, residing in different phase configurations, are likely to impact gene function and phenotype. Pair-wise genome comparisons were performed in the largest available set of 372EUR allowing a sufficiently large number of observations of identical pairs of mutations. In addition to identifying pairs of phase-different mutations at the whole genome level, we extracted the genes that had two identical mutations in both *cis* and *trans* configurations.

Supplementary Methods

Control of selection bias

To test whether sub-sampling of 57CEU-derived genomes may have introduced a selection bias, we computed the numbers of unique haplotypes and diplotypes in 10 randomly chosen subsets of 10 genomes and compared them to the initial haplotype/diplotype results. We obtained differences between 0.1 and 0.6% (0.4% on average) in the numbers of the computed unique haplotypes and diplotypes (see Table S7). The specific 57CEU-derived samples that had been selected for the described subsets of 5, 10, and 14 genomes

corresponded to the first 5, 10, and 14 samples of the 57CEU dataset listed in Supplementary Table 15. In the molecularly haplotype-resolved genomes, subset information was collected from the first 5 and 10 genomes out of the total of 14 where phasing information was available for a given gene.

Estimation of error in haplotype/diplotype quantification

A major source of potential error which could have an impact on the quantification of haplotypes and diplotypes is phase discordance. Any incorrectly phased heterozygous SNP (see comparative evaluation of molecular vs statistical phasing data) could introduce a false novel (unique) haplotype or diplotype.

To estimate the potential impact of phase discordance, note that the fraction of ~5% obtained represents a composite value, the sum of the error frequency in both fosmid-based molecular haplotyping. We have assessed a switch error rate of 1.69% for our fosmid pool-based haplotype assembly compared to gold standard trio data[10] (Duitama et al., 2012). For statistical phasing, a switch error rate in the range of ~2.5 – 3% was described[11] (Browning and Browning, 2011; the 1000 Genomes Project described a phasing (switch) error every 300-400 kb[7] (Abecasis et al., 2012). Thus, we evaluated the impact of phase discordance separately for the molecular and the statistical scenario, assuming a phase discordance of ~2% for molecular and of ~3% for statistical phasing. To this end, we analyzed in a first pass the original data set of 14 molecularly haplotype-resolved genomes (14G), which had been compared to their statistically inferred phase, to derive the phase discordance of ~5%. This allowed direct inspection of all existing phase discordant sites, which could result either from molecular or statistical switch errors. Accordingly, about 40% of these phase discordant sites could be attributed to a potential error in molecular phasing, and approximately 60% to a potential error in statistical phasing. To estimate the fraction of false novel molecular haplotypes, we examined every gene in each individual genome. In the case where 40% of the phase discordant SNPs in a gene would result in at least one phase discordant site (requiring at least 3 phase discordant SNPs), we scored (under assumption of a worst case scenario) a false pair of novel molecular haplotypes. Using this approach, we estimated that a fraction of 13.7% of the unique molecular haplotypes, and 10.6% of the unique diplotypes in 14G would be falsely considered novel due to a phasing (switch) error. The majority of these, 12.7% of the novel haplotypes and 9.8% of the novel diplotypes, were assigned to category 3 genes.

Inspecting the genes that contributed the fraction of 13.7% false novel molecular haplotypes more closely, we found that only a small fraction of those, 13% (all from category 3) contributed a disproportionately high amount of false novel haplotypes, each of their haplotypes appearing novel. These genes on average had 122 SNPs (median 52) and were found to contain numerous switched SNPs.

We used the original 14G data set analogously to estimate the fraction of false novel statistical haplotypes. Thus, in the case where 60% of the phase discordant SNPs in a gene were equal to at least one phase discordant site, we scored (assuming again a worst case scenario) a false pair of novel statistical haplotypes. We estimated that a fraction of 24.8% of the unique statistical haplotypes, and 18.9% of the unique statistical diplotypes were likely to be false novel due to a phasing (switch) error. 21.8% and 16.9% of those, respectively, were assigned to category 3 genes.

Most importantly, the errors are expected to be highly correlated, a non-random distribution of switch errors being an inherent feature of the methods applied. Thus, rare misassignment of a fosmid, or a similar mistake in assigning a region (e.g. LD block) in statistical haplotyping will affect a large number of heterozygous SNPs in parallel. (In a sense, giving the error rate on a per SNP basis is therefore somewhat misleading, and could be replaced by an estimate

of the frequency of misassignment of larger regions, and the average number of SNPs affected by such a misassignment.) High correlation of errors is in agreement with the large discrepancy between the observed data described above and the error estimates expected under random distribution. In the case of molecular phasing: 13.7% false novel haplotypes observed vs 43.9% expected; in the case of statistical phasing: 24.8% observed vs 48.8% expected (assuming that e.g. at a phase discordance of 3%, every gene with > 33 SNPs contributes false novel haplotypes).

In a second pass we estimated the fraction of false novel statistical haplotypes directly in 372EUR by use of probability calculations/measures. To assess the probability that a gene's haplotypes are incorrect, we applied the (only available) measure for phasing (switch) error provided by 1000G[7] (Abecasis 2012), the distance between switch errors given as 300 kb. Let *n* be the length of a gene and *k* the average 'median switch distance' per individual in kb (k=300, Abecasis 2012)[7], the probability P_G that the unique haplotypes of a gene are incorrect is defined as:

$$P_G\ (X = false) = \left(1 - \frac{1}{k}\right)^n = 0.996^n \qquad (1)$$

The numbers of potentially false novel haplotypes in 372EUR were calculated as follows:

Let *m* be the number of genes and *S* the singleton haplotypes of a gene, then the corresponding number of potentially false novel (unique) haplotypes in the sample is given by

$$FP_{haps} = \sum_{i=1}^{m} P_{G_i}\, S_i \qquad (2)$$

Accordingly we estimated that approximately 15% of the RefSeq genes contain false novel haplotypes in 372EUR, amounting to approximately 25% of all unique haplotypes.

Reassessing major results under consideration of these error estimations, the absolute numbers of unique haplotypes in 372EUR would potentially decrease from ~4.1 Mio to ~3.1 Mio, and the unique diplotypes from 3.9 Mio to 3.2 Mio; the fractions of unique haplotypes relative to total haplotype input count from 33.5% to 25.2%. The categorization of genes, that is, the relative fractions of category 1, 2 and 3 genes (see pie charts in Fig. 2) would change at most by 0.7%, and the analyses addressing protein sequences would remain essentially unaffected. Thus, this potential error fraction changes neither our key results on haplotype diversity nor our conclusions. In other words, the vast majority of the statistical haplotypes and diplotypes, ~75% and 81%, respectively, may be considered robustly quantified when scaling up the analysis. The conclusion that given phase discordance does not result in a major inflation of haplotypes is underscored by the observation that the fractions of unique singleton haplotypes/diplotypes were found to decrease (rather than increase) with increasing sample size (without any exception in all gene categories) up to 628 genomes from 1000G. This suggests that for the vast majority of singleton haplotypes, additional copies are detected with increasing sample depth.

Other potential sources of error impacting haplotype diversity: False positive (FP) and negative (FN) SNPs: As outlined above, 1000G uses mostly imputation to cope with missing genotypes. They describe FPs of ~1.7% and "no call" rates between 2.1 and 6%[7], suggesting as net effect an underestimation of novel haplotypes. Regarding the impact of false positive and negative SNPs (0.2 and 5.4% on average, respectively, see Table S2) on haplotype diversity in our molecularly haplotype-resolved genomes, the net effect will similarly result in an under-estimation of diversity. Combining all major sources of error, there seems to remain

an overestimation of approximately 10% (molecular) to 20% (statistical) of haplotype diversity.

Simulation studies consolidating the common diplotypic proteome

To address the reviewer's comment, we have conducted a simulation study based on a statistical argument. Recall, that we define a protein 'diplotypic' in a specific genome if it has at least one non-synonymous mutation in this genome. In order to assess, whether the observed frequency of a specific diplotypic protein in a population is higher than by chance we applied the Binomial test. Let *n* be the number of genomes under study and *p* the frequency for a diplotypic protein, then the probability of observing a protein exactly *k* times being diplotypic among the *n* genomes is

$$P(X = k) = \binom{n}{k} p^k (1-p)^{n-k} \quad (3)$$

and the corresponding P-value is

$$\sum_{i \geq k} P(X = i) \quad (4)$$

.

The probability, p, of a protein to encode a diplotype is assumed to be constant in every genome with p=0.18. This probability value was based on the observation that across all genomes, consistently between 16 and 22% of the autosomal genes (18% on average) were found to encode a protein diplotype (p. 10; Tables S5b and S14). We generated random data sets for sample sizes of 372 and 57 genomes (randomly scoring 18% of their autosomal genes as diplotypes). In the next step, we extracted from each data set a set of genes that were scored as protein diplotypes in at least 30% of the simulated genomes, the threshold defining the 'common diplotypic proteome' subset of genes. As a result, there were consistently zero genes found above this frequency threshold in 372 simulated genomes, and 162 (range of 152-170) genes in 57 genomes. The Binomial model was not run with the 14G scenario because of the low sample size. Our experimental observation yielded numbers that were far greater: 5,951 genes were found to have protein diplotypes in over 30% of the genomes in 372EUR, and 4,665 genes in 57CEU (Table S15a). This resulted in p-values of $p < 4.6x10^{-9}$ and $p < 9.3x10^{-3}$, respectively. Thus, in fact distinctive subsets of genes exist in given sample sets, which have the property 'diplotype' significantly more frequently compared to chance. These subsets were found to strongly overlap (~90%); the common diplotypic proteome integrating only genes contained within the overlap that is, having been observed in two independent samples.

Importantly moreover, our simulation studies did not result in generating any of the key features/data that provided the basis for our extraction of the common diplotypic proteome subset of genes:

1) Simulated graphs showing the distribution of diplotype frequencies across all autosomal genes were entirely different compared to Figs. 3a and S3, sorting the genes (alphabetically) by increasing diplotype frequencies: all genes had roughly similar diplotype counts, with only very few genes showing diplotype frequencies higher than the remainder and simulated diplotype frequencies far below observed frequencies; with this, lack of demonstration of subset nature;

2) Simulated data sets did not allow extraction of subsets of genes encoding protein diplotypes above defined frequency thresholds, as documented by the decreasing graphs in Fig. 3b (blue colors), and addressed in detail for the threshold of 30% above, which was used to define the common diplotypic proteome;

3) As a consequence, no substantial overlaps could be generated, if at all, between simulated gene sets at defined frequency thresholds (for comparison see orange and yellow graphs in Fig. 3b); specifically, at the frequency threshold of 30%, the overlap between 57 and 372 simulated genomes was zero (as compared to > 90% in real data), and the overlap between 14 and 57 simulated genomes was ~21% on average (as compared to > 83% in real data).

Taken together, none of the key steps/key data sets could be replicated by our simulation study that would result in the distinctive subsets of genes which we have integrated to a common diplotypic proteome.

Supplementary References

1 McKernan KJ, Peckham HE, Costa GL, McLaughlin SF, Fu Y, Tsung EF, Clouser CR, Duncan C, Ichikawa JK, Lee CC et al. 2009. Sequence and structural variation in a human genome uncovered by short-read, massively parallel ligation sequencing using two-base encoding. *Genome Res* **19**(9): 1527-1541.

2 Adzhubei, I. A. *et al.* A method and server for predicting damaging missense mutations. *Nature Methods* **7**, 248-249 (2010).

3 Ng, P. C. & Henikoff, S. Predicting deleterious amino acid substitutions. *Genome Res* **11**, 863-874 (2001).

4 Duitama, J., Huebsch, T., McEwen, G., Suk, E.-K. & Hoehe, M. R. in *Proceedings of the First ACM International Conference on Bioinformatics and Computational Biology* 160-169 (ACM, Niagara Falls, New York, 2010).

5 Abecasis, G. R. *et al.* A map of human genome variation from population-scale sequencing. *Nature* **467**, 1061-1073 (2010).

6 Scheet, P. & Stephens, M. A fast and flexible statistical model for large-scale population genotype data: applications to inferring missing genotypes and haplotypic phase. *Am J Hum Genet* **78**, 629-644 (2006).

7 Abecasis, G. R. *et al.* An integrated map of genetic variation from 1,092 human genomes. *Nature* **491**, 56-65 (2012).

8 Young, M. D., Wakefield, M. J., Smyth, G. K. & Oshlack, A. Gene ontology analysis for RNA-seq: accounting for selection bias. *Genome Biol* **11**, R14 (2010).

9 Fox-Walsh KL, Dou Y, Lam BJ, Hung SP, Baldi PF, Hertel KJ. The architecture of pre-mRNAs affects mechanisms of splice-site pairing. *Proceedings of the National Academy of Sciences of the United States of America* **102**, 16176-16181 (2005).

10 Duitama, J. *et al.* Fosmid-based whole genome haplotyping of a HapMap trio child: evaluation of Single Individual Haplotyping techniques. *Nucleic Acids Res* **40**, 2041-2053 (2012).

11 Browning, S. R. & Browning, B. L. Haplotype phasing: existing methods and new developments. *Nat Rev Genet* **12**, 703-714 (2011).
